# The molecular function of kallikrein‐related peptidase 14 demonstrates a key modulatory role in advanced prostate cancer

**DOI:** 10.1002/1878-0261.12587

**Published:** 2019-11-28

**Authors:** Thomas Kryza, Nathalie Bock, Scott Lovell, Anja Rockstroh, Melanie L. Lehman, Adam Lesner, Janaththani Panchadsaram, Lakmali Munasinghage Silva, Srilakshmi Srinivasan, Cameron E. Snell, Elizabeth D. Williams, Ladan Fazli, Martin Gleave, Jyotsna Batra, Colleen Nelson, Edward W. Tate, Jonathan Harris, John D. Hooper, Judith A. Clements

**Affiliations:** ^1^ Australian Prostate Cancer Research Centre‐Queensland (APCRC‐Q) Institute of Health & Biomedical Innovation Queensland University of Technology Woolloongabba Australia; ^2^ School of Biomedical Sciences Faculty of Health Queensland University of Technology Woolloongabba Australia; ^3^ Translational Research Institute Woolloongabba Australia; ^4^ Mater Research Institute – The University of Queensland Brisbane Australia; ^5^ Department of Chemistry Imperial College London UK; ^6^ Vancouver Prostate Centre Department of Urologic Sciences University of British Columbia Canada; ^7^ Faculty of Chemistry University of Gdansk Poland; ^8^ Mater Health Services South Brisbane Australia

**Keywords:** castrate‐resistant prostate cancer, kallikrein‐related peptidase, metastasis, prostate cancer, protease, protease‐substrate

## Abstract

Kallikrein‐related peptidase 14 (KLK14) is one of the several secreted KLK serine proteases involved in prostate cancer (PCa) pathogenesis. While relatively understudied, recent reports have identified KLK14 as overexpressed during PCa development. However, the modulation of KLK14 expression during PCa progression and the molecular and biological functions of this protease in the prostate tumor microenvironment remain unknown. To determine the modulation of KLK14 expression during PCa progression, we analyzed the expression levels of KLK14 in patient samples using publicly available databases and immunohistochemistry. In order to delineate the molecular mechanisms involving KLK14 in PCa progression, we integrated proteomic, transcriptomic, and *in vitro* assays with the goal to identify substrates, related‐signaling pathways, and functional roles of this protease. We showed that KLK14 expression is elevated in advanced PCa, and particularly in metastasis. Additionally, KLK14 levels were found to be decreased in PCa tissues from patients responsive to neoadjuvant therapy compared to untreated patients. Furthermore, we also identified that KLK14 expression reoccurred in patients who developed castrate‐resistant PCa. The combination of proteomic and transcriptomic analysis as well as functional assays revealed several new KLK14 substrates (agrin, desmoglein 2, vitronectin, laminins) and KLK14‐regulated genes (Interleukin 32, midkine, SRY‐Box 9), particularly an involvement of the mitogen‐activated protein kinase 1 and interleukin 1 receptor pathways, and an involvement of KLK14 in the regulation of cellular migration, supporting its involvement in aggressive features of PCa progression. In conclusion, our work showed that KLK14 expression is associated with the development of aggressive PCa suggesting that targeting this protease could offer a novel route to limit the progression of prostate tumors. Additional work is necessary to determine the benefits and implications of targeting/cotargeting KLK14 in PCa as well as to determine the potential use of KLK14 expression as a predictor of PCa aggressiveness or response to treatment.

## Graphical abstract

Kallikrein‐related peptidase 14 (KLK14) is a secreted serine protease belonging to the kallikrein‐related‐peptidase family. We identified that KLK14 is overexpressed in advanced prostate cancer and acts on various substrates involved in adhesion, migration, and invasion of cancer cells, thus modulating genes and signaling pathways essential for prostate cancer progression. These results suggest that KLK14 is a potential target to control aggressiveness of prostate tumors.

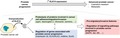

**Abbreviations**
AGRN: agrin
AR: androgen receptor
ATT: androgen‐targeting therapies
CM: conditioned medium
CRPC: castrate‐resistant prostate cancer
CSS: charcoal‐stripped serum
DFP: disease‐free progression
DHT: dihydrotestosterone
ECM: extracellular matrix
IHC: immunohistochemistry
IPA: ingenuity pathway analysis
KLK: kallikrein‐related peptidase
MAPK: mitogen‐activated protein kinase
NHT: neoadjuvant hormonal therapy
OBM: osteoblast‐derived 3D microtissue
PCa: prostate cancer
PSA: prostate‐specific antigen
RTqPCR: reverse‐transcription quantitative polymerase chain reaction
TAILS: terminal amine isotopic labeling of substrates
TCGA: The Cancer Genome Atlas

## 1 Introduction

Prostate cancer (PCa) is the second most frequent nonskin cancer diagnosed in developed countries. The 5‐year survival of patients diagnosed with localized PCa is close to 100%; however, recurrent and/or metastatic PCa remains incurable and is responsible for most PCa‐related deaths (Pascale *et al.*, 2017). The main treatment strategies for advanced PCa focus on the dependency of PCa cells on androgens and work by inhibition of the androgen receptor (AR) axis by blocking androgen production (i.e., degarelix, abiraterone) or direct targeting of the AR (i.e., bicalutamide, enzalutamide) (Crona and Whang, 2017). To date, these strategies have been very effective and significantly increase the survival of PCa patients. Nevertheless, the cancer cells may eventually adapt to, and overcome, androgen‐targeting therapies (ATT) and these patients will eventually develop castrate‐resistant PCa (CRPC) and metastatic disease (Crona and Whang, 2017). Identification of the molecular factors involved in the progression of PCa cells during ATT is critical to address this issue and could lead to the development of new therapeutic agents to help prevent recurrence at this stage.

Proteases are well‐known for their functional involvement in cancer progression exerting either tumor‐promoting or tumor‐suppressing effects (López‐Otín and Matrisian, 2007; Vasiljeva *et al.*, 2019). Particularly, proteases are involved in several essential cellular events required for cancer cells to disseminate from a primary tumor to a secondary site where cancer cells establish metastasis (Filippou *et al.*, 2016; Sleeboom *et al.*, 2018). The kallikrein‐related peptidases (KLKs) are a family of fifteen secreted serine proteinases which are well‐known for their involvement in cancer progression (Filippou *et al.*, 2016; Kryza *et al.*, 2016). KLK14, a kallikrein exerting trypsin‐like proteolytic activity, is the focus of several research groups because of its possible involvement in several pathologies, such as skin defects and hormonal cancers. The physiological role of KLK14 is not clearly determined. KLK14 is expressed in skin where it could be involved in epidermis physiology as it is able to activate the main effectors of skin desquamation KLK5 and KLK7 as well as to directly degrade the desmoglein‐1 protein *in vitro* (Furio *et al.*, 2014; Furio *et al.*, 2015; Yoon *et al.*, 2007). KLK14 is also detected in normal breast and prostatic tissues, but its functions in those tissues have not been yet determined (Hooper *et al.*, 2001; Kryza *et al.*, 2016; Rajapakse and Takahashi, 2007; Yousef *et al.*, 2003). The dysregulation of KLK14 expression has also been observed in other malignancies such as in high‐grade serous ovarian cancer, where it plays a protective role through an unidentified mechanism (Dettmar *et al.*, 2018) and in colon cancer, where it promotes tumorigenesis through activation of proteinase‐activated receptors (Chung *et al.*, 2012; Devetzi *et al.*, 2013). Moreover, to date, several studies have demonstrated that KLK14 expression is elevated in PCa compared to normal prostatic tissues. Furthermore, high KLK14 expression is associated with indicators of poor patient outcome, notably prostate‐specific antigen (PSA/KLK3) detected biochemical relapse (Borgono *et al.*, 2007b; Lose *et al.*, 2012; Rabien *et al.*, 2008; Reid *et al.*, 2016; Yousef *et al.*, 2003). The association of KLK14 with PCa is also supported by genetic studies which showed that polymorphisms in the *KLK14* gene are significantly associated with PCa aggressiveness (Lose *et al.*, 2012; Rose *et al.*, 2018).

However, to date, any variation in the expression levels of KLK14 during PCa progression after neoadjuvant hormonal therapy (NHT) has not been determined, and the molecular mechanisms supporting its protumorigenic effect in the prostate tumor microenvironment remain elusive. To assess whether KLK14 levels change during PCa progression, we analyzed KLK14 mRNA expression in advanced PCa using publicly available databases and KLK14 protein expression in PCa tissues obtained from untreated patients, from those responding to NHT and from patients with CRPC. In order to elucidate the molecular functions of KLK14 in the prostate tumor microenvironment, we employed proteomic and transcriptomic pathway analysis, and functional approaches and comprehensively identified the KLK14 substratome, associated downstream pathways, and its cellular functions.

## 2 Materials and methods

### 2.1 Chemicals, reagents, and antibodies

All reagents and materials were purchased in Australia except when indicated. Cell culture reagents were sourced from Gibco (ThermoFisher, North Ryde, Australia) and chemical reagents were purchased from Sigma (Castle Hill, Australia) except when specified. Antibodies were purchased from the following vendors: anti‐KLK14 catalytic domain antibody (Triple Point Biologics, Forest Grove, OR, USA), anti‐beta actin and anti‐GAPDH (mouse monoclonal; Abcam, Cambridge, UK), anti‐KLK3/PSA (rabbit polyclonal; Dako, North Sydney, Australia), anti‐agrin (AGRN; rabbit polyclonal; ThermoFisher), anti‐phospho Erk1/2 (Tyr202/204, rabbit polyclonal; Cell Signaling Technology, Genesearch, Arundel, Australia), anti‐Erk1/2 (mouse monoclonal; Cell Signaling Technology), anti‐phospho Mek1/2 (Ser217/221, rabbit monoclonal; Cell Signaling Technology), anti‐Mek1/2 (mouse monoclonal; Cell Signaling Technology), and anti‐GFP (mouse monoclonal; Abcam). Goat anti‐mouse IgG Alexa Fluor‐680, goat anti‐rabbit IgG Alexa Fluor‐680, goat anti‐rabbit IgG Alexa Fluor‐488, and Alexa Fluor‐647 Phalloidin were purchased from Thermo Fisher Scientific (Seventeen Mile Rocks, Australia). Recombinant human KLK14 (2626‐SE), purified thermolysin (3097‐ZN), recombinant human AGRN (6624‐AG), recombinant human desmoglein‐2 (DSG2; 947‐DM), and recombinant human cystatin‐C (CST3; 1196‐PI) were purchased from R&D Systems (In Vitro Technologies, Noble Park, Australia). The SFTI‐WCIR was produced as previously described (de Veer *et al.*, 2017).

### 2.2 Evaluation of KLK14 expression in clinical samples

For publicly available microarray expression datasets, the normalized expression data for KLK14 was downloaded from the Oncomine database (http://www.oncomine.org). For publicly available gene expression data from The Cancer Genome Atlas (TCGA), the normalized expression data (count + 1) for KLK14 and KLK3/PSA was downloaded from the University of California Santa Cruz Xenabrowser (https://xenabrowser.net/).

### 2.3 Immunohistochemistry

An NHT and CRPC tissue microarray comprising samples from 420 PCa patient samples were obtained from the Vancouver Prostate Centre Tissue Bank. Specimens were obtained from patients following informed written consent using a protocol conformed to the standard set by the Declaration of Helsinki and approved by the Clinical Research Ethics Board of the University of British Columbia (BC) and the BC Cancer Agency. Immunohistochemistry (IHC) was performed using a rabbit monoclonal antibody against the human KLK14 catalytic site (Triple Point Biologics, 1 : 500 dilution) using a Ventana Discovery XP Autostainer and Ventana/Roche (West End, Australia) reagents. Briefly, antigen retrieval was performed using extended CC1 solution. The primary antibody was incubated for 60 min and the secondary antibody (anti‐rabbit HQ) was incubated for 8 min, followed by tertiary reagent anti‐HQ. Slides were counterstained using hematoxylin and eosin. Evaluation of the staining intensity was performed accounting for the percent of cancer tissue stained (*N*) and the intensity of staining (*I*) and score for each sample were calculated using the formula N*I/100.

### 2.4 Human cell lines

Human PCa cell lines (LNCaP, C4‐2B, DU145, and PC‐3) were sourced from the American Type Culture Collection (Manassas, VA, USA). Cells were used in passages 17–37, authenticated, and regularly tested for mycoplasma infection. Cell lines were cultured routinely in RPMI‐1640 phenol‐red free medium + l‐glutamine containing 5% FBS (Gibco). Culture medium composition was modified for some experiments: in androgen stimulation/inhibition experiments, FBS was replaced by charcoal stripped FBS (CSS) to limit the amount of androgens supplied by serum; for culture of LNCaP cells transduced with pLIX302, culture medium was supplemented with 1 μg·mL^−1^ puromycin; in experiments to investigate the effect of KLK14 proteolytic activity, the amount of FBS/CSS was reduced to 1% to limit the amount of protease inhibitors within the FBS; in experiments involving an analysis of conditioned medium (CM), cells were grown in serum‐free medium.

### 2.5 Bioengineered cell lines

To generate inducible‐KLK14‐LNCaP cells (iKLK14‐LNCaP), the coding sequence of human pre‐pro‐KLK14 (UniProt accession: http://www.uniprot.org/uniprot/Q9P0G3, CCDS12823.2) was cloned into the pLIX402 vector (tetracycline‐inducible lentiviral expression; Addgene, Watertown, MA, USA, #41394). To generate inducible catalytically inactive KLK14 (imKLK14), the codon encoding the catalytic serine at position 195 was mutated to encode an alanine (S195A). As a control, the pLIX402 vector containing GFP sequence was used. To generate expression vectors encoding mKLK14‐GFP and KLK14‐GFP, the STOP codon in the KLK14 coding sequence was deleted to allow the addition of a GFP‐tag at the C terminus of KLK14. Lentiviral particles generated in HEK293‐Freestyle cells were used to transduce LNCaP cells which were subsequently selected with puromycin. To generate mKO2‐LNCaP cells, cells were transduced using the pLEX307 (constitutive lentiviral expression, Addgene #41392) plasmid containing the coding sequence of mKO2 protein and highly fluorescent cells were sorted using an Astrios cell sorter (Beckman Coulter, Lane Cove West, Australia).

### 2.6 Androgen and anti‐androgen treatments

Prostate cancer cells were seeded in growth medium before medium was replaced with RPMI containing 5% CSS 48 h later. After 48 h, medium was replaced with fresh RPMI/5% CSS and cells were treated with 10 μm enzalutamide (ENZ, Selleck Chemicals, Sapphire Bioscience, Redfern, Australia) in the presence of 10 nm dihydrotestosterone (DHT) or ethanol vehicle control for 5 days. Media and treatments were replaced every 48 h.

### 2.7 Western blotting

Cell lysate protein extracts were produced using RIPA buffer (Sigma) supplemented with sodium orthovanadate, sodium fluoride, and EDTA‐free protease inhibitor cocktail (Roche). To prepare protein extracts from CM, cell supernatants were spun at 2000 ***g*** for 10 min at 4 °C before filtration (0.2 µm syringe filters) and concentration using Amicon Ultra‐15 Centrifugal Filter Units (3 kDa cut off). Protein concentration was determined by the BCA (Pierce, Thermo Fisher Scientifc). Equal amounts of protein were resolved on 4–12% NuPAGE gels with 3‐(*N*‐morpholino)‐propanesulfonic acid running buffer (ThermoFisher). Resolved proteins were transferred onto nitrocellulose membrane by liquid transfer performed with Tris/glycine SDS buffer containing 20% of methanol. Protein detection was performed on a Li‐Cor Odyssey system after incubation with primary antibody diluted at 1 : 1000 in Tris‐buffered saline (TBS) solution containing 0.1% Tween‐20 and 5% BSA overnight at 4 °C and with secondary antibody diluted at 1 : 20 000 in the same buffer 1h at RT.

### 2.8 Active‐KLK14 NeutrAvidin Blot using activity‐based probe

Conditioned media (10 μg) was treated with the biotinylated activity‐based probe (compound X, Fig. S1B) for 2 h. After 2 h, 3 μL of sample loading buffer was added and the sample was boiled for 5 min. Aliquots were then loaded on to SDS/PAGE Gels and ran for 10 min at 80 volts and 50 min at 180 volts. Proteins were transferred to a nitrocellulose membrane (GE Healthcare, Mansfield, Australia, Hybond ECL, pore size 0.45 μm) using a wet transfer setup and a Tris/glycine transfer buffer supplemented with 20% MeOH. Membranes were washed with TBS‐T (1 × TBS, 0.1 % Tween‐20), blocked for 1 h with a 3 % BSA solution in TBS‐T, and incubated with NeutrAvidin‐HRP (1 : 1000 dilution in 0.3 % BSA) for 1 h. After 1 h, the membrane was washed with TBS‐T (3 × 10 min) and then developed with Luminata Crescendo Western HRP substrate (Millipore, Macquarie Park, Australia), according to the manufacturer’s instructions.

### 2.9 Proteolytic activity assay

Kallikrein‐related peptidase 14 proteolytic activity was measured in concentrated CM prepared as above using a FRET substrate designed to be specific to KLK14 (ABZ‐Y‐G‐P‐R‐V‐L‐P‐Y‐(NO2)‐NH2). Briefly, 90 µL of concentrated CM was incubated with 10 µL of FRET substrate (500 µm) in a black 96‐well per plate and fluorescence emission was followed using a PHERAstar plate reader (BMG labtech, Mornington, Australia, wavelength excitation = 320 nm, wavelength emission = 450 nm) as soon as the substrate was added. Results are shown as the averages of *n* = 3 samples subtracted from blank values.

### 2.10 Transcriptome analysis

Analysis of an Agilent 4 × 180 k custom oligoarray of triplicate samples was performed as described (Wang *et al.*, 2018). Total RNA was extracted with an ISOLATE II RNA kit (Bioline, Eveleigh, Australia). RNA samples were screened with an Agilent Bioanalyzer to ensure the RNA was of high quality, and 100 ng was amplified and labeled following the manufacturer's instructions for One‐Color Microarray‐based Gene Expression Analysis (Agilent, Mulgrave, Australia). Microarray data were processed with Agilent's feature extraction software (v.10.7), and differential expression was determined using a Bayesian adjusted t‐statistic within the ‘Linear Models for Microarray Data’ r‐package. *P* values were corrected for a false discovery rate (FDR) of 5%. Probes with a fold change (FC) of ≥ 1.5/≤ −1.5 and FDR corrected *P* ≤ 0.05 between two groups were defined as significantly different.

### 2.11 Proteomic analysis

#### 2.11.1 Isobaric labeling and depletion of trypsin‐generated neo‐N termini

imKLK14‐ and iKLK14‐LNCaP cells were grown until 50% confluent in normal medium. After several gentle washes in PBS, cells were incubated overnight in serum‐free RPMI before medium was replaced with serum‐free RPMI containing 100 ng·mL^−1^ doxycycline. After 48 h, CM was harvested and concentrated using 3 kDa filter columns. Samples in biological triplicates were prepared as described (Munasinghage Silva *et al.*, 2019) with a few modifications as given below. The labeling reaction was performed with TMTduplex™ Isobaric label reagents (TMT^126^ and TMT^127^; ThermoFisher Scientific) in 50% v/v DMSO (at 25 °C 1 h in the dark) and quenched by adding 100 mm ethanolamine and vortexing (RT, 30 min). Isobaric tags used were inverted between conditions for each biological replicate. The same amounts of proteins from each condition were pooled and trypsin digested. A fraction of each trypsin‐digested sample (10%) was preserved for preterminal amine isotopic labeling of substrates (Pre‐TAILS) analysis while 90% of the peptide samples were depleted of trypsin‐generated neo‐N Termini using the HPG‐ALD polymer (available through ://www.flintbox.ca). Unbound peptides were eluted from HPG‐ALD polymer using Amicon Ultra‐0.5 Centrifugal Filter Units (10 kDa cut off). Peptide samples (Pre‐TAILS and TAILS) were desalted using C18 StageTips, which were preconditioned with 100 µL of 100% acetone. Any remaining organic solvent was removed from the column with 100 µL of buffer A (0.1% formic acid in milliQ water (Sigma) or acidified water) twice. The acidified peptide sample (pH < 3.5) was forced through the column three times followed by two washes with buffer A. Peptides were eluted with 40 µL of buffer B (0.1% formic acid, 80% acetonitrile in water), vacuum dried, and redissolved in 0.1% formic acid by sonicating for 10 min and spin‐vortexing for another 10 min. The mass spectrometry N‐terminomics data have been deposited to the ProteomeXchange Consortium via the PRIDE partner repository with the dataset identifier PXD015128.

#### 2.11.2 LC‐MS/MS analysis

The resulting peptides were analyzed by LC‐MS/MS using the Easy LC‐Q Exactive Plus system (Thermo Scientific, Bremen, Germany). Samples were loaded on a nanoViper PepMap100 C18 trap column (75 μm × 2 cm) in 3% (v/v) acetonitrile/ 0.1% (v/v) formic acid, at a flow rate of 20 μL·min^−1^. Peptides were eluted and separated at a flow rate of 250 nL·min^−1^ on a PepMap C18 nanocolumn (2 μm 100 Å pore size, 75 μm × 50 cm; Thermo Scientific), where acetonitrile was elevated from 3% (v/v) to 25% (v/v) over 60 min, followed with a linear acetonitrile gradient from 25% (v/v) to 40% (v/v) in 0.1% (v/v) formic acid for 12 min, followed by a linear increase to 95% (v/v) acetonitrile in 0.1% (v/v) formic acid over 1 min, followed by 15 min of elution with 95% (v/v) acetonitrile in 0.1% (v/v) formic acid and by reduction of acetonitrile back to 3% (v/v)and re‐equilibration. The eluent was nebulized and ionized using a Thermo nano electrospray source with a distal coated fused silica emitter (New Objective, Woburn, MA, USA). Typical mass spectrometric conditions were as follows: spray voltage, 1.8 kV; no sheath and auxiliary gas flow; heated capillary temperature, 250 °C.

The Q Exactive instrument was operated in the data‐dependent mode to automatically switch between full‐scan MS and MS/MS acquisition. Survey full‐scan MS spectra (*m*/*z* 350–1400) were acquired in the Orbitrap with 70 000 resolution (*m*/*z* 200) after accumulation of ions to a 3 × 10^6^ target value with maximum injection time of 50 ms. Dynamic exclusion was set to 30 s. The 10 most intense multiple charged ions (*z* ≥ 2) were sequentially isolated and fragmented in the octopole collision cell by higher energy collision dissociation (HCD, normalized collision energy 32%) with a maximum injection time of 100 ms. MS/MS spectra were acquired in the Orbitrap with 35 000 resolution, automatic gain control target of 1 × 10^5^ counts, and 1.2 *m*/*z* isolation width. Underfill ratio was at 1%.

#### 2.11.3 Data analysis

Raw files generated were analyzed using thermo proteome discoverer 2.2.0.388 software (Thermo Fisher Scientifc) against the UniProt Knowledgebase (UniProtKB) human protein database. Reverse sequences (Decoys) informed false positive identification frequency. Search parameters were as follows: precursor ion mass (monoisotopic) tolerance ± 10 p.p.m.; MS/MS tolerance ± 0.02 Da; and semi‐Arg‐C cleavage allowing for up to two missed cleavages. Fixed modifications included C carbamidomethylation (+57.021) and TMT2plex (+225.156) of K residues. Variable modifications included M oxidation (+15.995), acetylation (+42.011) of N termini, and TMT2plex of N termini (+225.156). Only peptides identified in two PSM and in two biological replicates, quantified, not marked as contaminant, and with a percolator *q*‐value (Sequest HT) and FDR < 0.01 were considered for subsequent analysis. For the quantification analysis, mean for the log2(iKLK14 : imKLK14) values and *P*‐value from ANOVA analysis were used. Histogram of the log2 ratios was plotted to check for normal distribution of proteins and peptides identified in Pre‐TAILS analysis. The standard deviation of the log2 ratios for Pre‐TAILS identified peptides was calculated and 2SD was used as cut off to determine the peptides with significant quantitative variations in Pre‐TAILS and TAILS analysis.

### 2.12 Proteolysis assay

Activation of recombinant human KLK14 was performed as recommended per the manufacturer using thermolysin. For dose–response assay, substrate proteins were incubated with an increasing amount of active KLK14 (molar ratio of enzyme/substrate of 1 : 5, 1 : 100) in assay buffer [50 mm Tris, 150 mm NaCl, 0.05% (w/v) Tween‐20, pH 8.0] for 2h. For kinetic assay, substrate proteins were incubated with active KLK14 at a molar ratio of enzyme/substrate of 1 : 50 for 5, 15, 30, 60, and 120 min. The proteolysis reactions were stopped by adding Laemmli loading buffer containing 5% of β‐mercaptoethanol. As a control, substrates and the highest concentration of active KLK14 used were incubated alone for the same period of time. SDS/PAGE and silver staining were performed as previously described.

### 2.13 Immunofluorescence staining

Cells were washed with PBS and fixed with 4% paraformaldehyde (PFA) in PBS for 15 min at RT. After washes, cells were permeabilized with PBS containing 0.1% of Triton X100 for 5 min before incubation with PBS + 3% BSA (w/v) for 1 h at RT. After washes with PBS, cells were incubated with PBS containing phalloidin at 1 : 40 and DAPI at 1 : 1000 for 30 min at RT. For AGRN staining, cells were incubated overnight at 4 °C with PBS + 1% BSA + anti‐AGRN antibody (1 : 200); after washes with PBS, cells were incubated with goat anti‐rabbit IgG Alexa Fluor‐488 diluted at 1 : 2000 in PBS + 1% BSA. Imaging was performed with a spinning disk confocal and maximum intensity projections were reconstructed using the Imaris software.

### 2.14 Quantitative PCR

Selected significantly regulated genes were validated by performing quantitative reverse‐transcription quantitative PCR (RT‐PCR) (Loessner *et al*., 2019) using gene‐specific primer pairs (Table S1). Total RNA extracted was reverse transcribed with SuperScript III Reverse Transcriptase (Thermo Fisher Scientific). Quantitative PCR was performed using SYBR Green (Applied Biosystems, Mulgrave, Australia) on a ViiA 7 Real‐Time PCR System (Applied Biosystems). Gene expression was determined by the comparative Ct method and normalized to the housekeeping gene (7SL and/or RPL32). Primer sequences used are listed in Table S1.

### 2.15 Proliferation and 2D migration assays

For proliferation assays, 5000 LNCaP cells or 3000 PC3 cells were seeded overnight into 96‐well plates (Corning, In vitro Technologies, Eight Mile Plains, Australia). Cells were washed in PBS, and fresh medium containing 1% FBS or 1% CSS was added. After 72 h, proliferation was assessed using CellTiter AQueous One Solution Reagent (Promega, Hawthorn, Australia). For migration assays, 20 000 LNCaP cells or 15 000 PC‐3 cells were seeded in a 96‐well Image‐lock plate (Essen BioScience Inc., Ann Arbor, MI, USA). Wounds were made through the monolayer of confluent cells using the 96‐pin WoundMaker (Essen BioScience Inc.) according to the manufacturer’s instructions. Wells were washed twice with PBS and fresh medium (RPMI + 1% FBS) was added before the initiation of imaging. Images were captured every 2 h for up to 72 h by the IncuCyte FLR live cell imaging system (Essen BioSciences Inc.). Wound closure kinetics were determined using the CellPlayer software module (Essen BioScience Inc.).

### 2.16 Preparation of osteoblast‐derived mineralized 3D microtissues

Osteoblast‐derived 3D microtissue (OBM) were prepared according to Bock *et al. *(2019). Briefly, medical grade polycaprolactone microfiber scaffolds were manufactured by melt electrowriting, using a custom in‐house built apparatus (IHBI, QUT, Brisbane, Australia), as per established protocols. The resulting biomaterial print had the following average dimensions: 500 µm thickness, 12 µm fiber diameter, and 150 µm pore size, and was cut in 1 × 1 cm scaffolds and coated with calcium phosphate (CaP) coating prior to sterilization for cell seeding. Isolation of human osteoprogenitor cells from donor bone tissue was performed in accordance with QUT ethics approval number 1400001024. Isolated cells were seeded at passage 4–5 on sterilized scaffolds (0.4 × 10^6^ cells per scaffold) and differentiated osteogenically for 8 weeks. Osteoblast phenotype was confirmed by mineral deposition and typical bone markers at gene and protein levels and reported here (Bock *et al.*, 2019).

### 2.17 Colonization of OBM

The OBM constructs containing live human osteoblasts and mineralized matrix (two patients, (Bock *et al.*, 2019)) were washed in RPMI and 5% FBS before coculture with LNCaP cells. The 1 × 1 cm live OBM constructs were cut in three identical stripes before coculture with the iGFP‐, imKLK14‐, and iKLK14‐LNCaP‐mKO2 cells for migration analysis. Randomized OBM stripes were placed in agar‐coated 24‐well plate, and 50 000 cells were seeded onto OBM in RPMI‐5% FBS containing 100 ng·mL^−1^ of doxycycline and incubated (37 °C, 5% CO_2_) overnight on a rocking mixer platform. The constructs were washed 3 times with SF‐RPMI, and placed in an uncoated 24‐well plate with fresh RPMI‐1% FBS containing doxycycline. Teflon rings were used to secure the scaffold during the live cell imaging (Olympus BX60 microscope, Cy3 filter and bright field, objective 4×, imaging at intervals of 20 min for 48 h). Videos were reconstructed from images (145 frames in total). An average of eight fields of view was recorded per construct. Mean square displacement (MSD), speed, and straightness were determined by automated spots statistics using Imaris (algorithm parameters: estimated cell diameter 18 μm, intensity filter 30–230, max distance jumps 20 μm, max gap size 5). For the colonization analysis, at the end of the migration analysis, the OBM‐LNCaP coculture constructs were cultured for a further 8 days (10 days in total) in RPMI‐1% FBS containing 100 ng·mL^−1^ doxycycline with media change every 3 days. The OBM‐LNCaP coculture constructs were washed with PBS, fixed in 4% PFA (3 h), and cells were permeabilized (Triton X100, 0.05% in PBS). After DAPI and phalloidin staining, the entire constructs were imaged with an inverted IX73 epifluorescent microscope (objective 2×). Image reconstruction was performed with imagej using the stitching function. The area occupied by LNCaP cells (mKO2 tagged) was measured using the area measurement function of imagej (four replicates per condition).

### 2.18 Statistical analysis

All statistical tests, other than microarray and mass spectrometry, were performed in graphpad prism (V7.00) (San Diego, CA, USA). Significance level was determined at *P < 0.05, **P < 0.01, ***P < 0.001, and ****P < 0.0001. For biological and biochemical experiments, unless otherwise stated, three independent experiments were conducted with mean ± standard error presented. For comparison between two groups, Mann–Whitney test was performed while Kruskal–Wallis test was performed for comparison between more than two groups.

## 3 Results

### 3.1 KLK14 expression is elevated in advanced PCa

**Figure 1:**
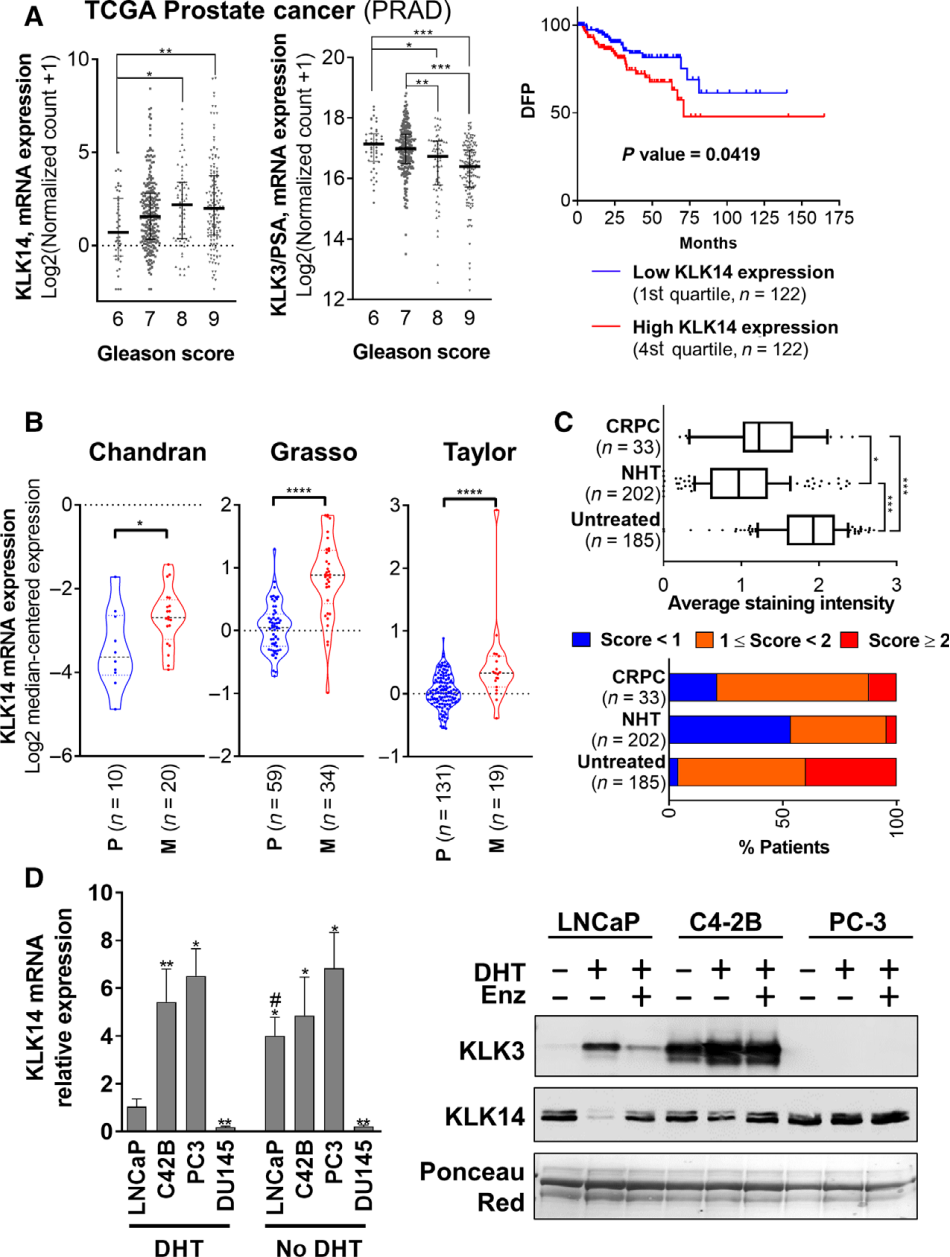
KLK14 expression is associated with advanced PCa. (A) Expression of KLK14 and KLK3/PSA mRNA in the TCGA prostate adenocarcinoma dataset. Left: Expression of KLK14 mRNA increases with Gleason score of PCa. KLK3/PSA mRNA is shown for comparison. Results are presented as median ± interquartile range. Right: Patients with high levels of KLK14 mRNA in the primary tumor (4th quartile, 122 samples) have a significant shorter DFP compared to patients with low levels of KLK14 expression in the primary tumor (1st quartile, 122 samples). (B) Expression of KLK14 mRNA in PCa‐derived metastases (Chandran, Grasso, Taylor datasets, Oncomine data portal; median ± interquartile range). KLK14 mRNA is significantly elevated in PCa‐derived metastases (M) compared to primary tumor (P). (C) Top: Average KLK14 expression determined by IHC on primary tumors from 185 untreated patients, 202 patients responsive to neoadjuvant hormonal therapy (NHT), and in 33 patients who developed CRPC. Results are presented as median ± interquartile range and 10–90 percentile. Bottom: Percentage of samples scored with low (< 1), medium (1 ≤ score < 2) and high (score > 2) staining per patient groups. (D) Left: Expression of KLK14 (mRNA level, RTqPCR, mean ± SD) in androgen‐dependent LNCaP cells and androgen‐independent and metastatic PCa cells (C42B, PC‐3 and DU145 cells) grown in CSS ± 10 nm DHT for 48h. *N* = 3, *P < 0.05, **P < 0.01, ***P < 0.001, compared to LNCaP cells DHT condition, ^#^ *P* < 0.05, compared to the respective DHT condition for each cell line. Two‐way ANOVA test was performed. Right: KLK14 and KLK3/PSA protein expression (western blot) in concentrated conditioned media from LNCaP, C4‐2B, and PC‐3 cells grown in serum‐free medium ± 10 nm DHT ± 10 µm Enz (enzalutamide) for 72 h.

Analysis of the prostate adenocarcinoma TCGA dataset indicated that KLK14 mRNA expression was significantly higher in high Gleason score (8 and 9) PCa compared to low Gleason score (6) PCa (Fig. 1A left). In contrast, the expression of KLK3 (PSA) was significantly lower in high Gleason score tumors in the same intergroup comparisons (Fig. 1A middle). Moreover, patients with high KLK14 mRNA expression (fourth quartile, *n* = 122) have significantly shorter disease‐free progression (DFP, *P* = 0.0419) compared to patients with low KLK14 expression (first quartile, *n* = 122, Fig. 1A right). The mRNA expression levels of KLK14 were further compared between primary PCa tumors and metastases in three independent datasets with at least 10 samples per condition (Oncomine (Rhodes *et al.*, 2004)). *KLK14* expression was significantly higher in metastasis compared to primary PCa in the three datasets analyzed (Chandran Prostate (Chandran *et al.*, 2007), *P* = 0.0311; Grasso Prostate (Grasso *et al.*, 2012), *P* < 0.0001; Taylor Prostate 3 (Taylor *et al.*, 2010), *P* = 0.0002, Fig. 1B). The level of KLK14 protein expression was also analyzed by IHC in tissue microarrays with 420 samples from PCa patients either untreated (*n* = 185), treated and responsive to NHT (*n* = 202) or who developed CRPC (*n* = 33; Fig. 1C and Fig. S1A). The KLK14 protein levels were significantly lower in samples from patients treated with NHT compared to samples from untreated patients but increased again in patients who developed CRPC (Fig. 1C top). When stratified with respect to staining intensity, a higher proportion of CRPC patients have high or intermediate KLK14 expression (78%) compared to NHT patients (47%; Fig. 1C bottom), suggesting that KLK14 expression decreases in patients responsive to NHT but reoccurs in patients developing CRPC.

We further investigated KLK14 expression in a range of low‐metastatic and androgen‐dependent (LNCaP) and high‐metastatic androgen‐independent (C4‐2B, PC‐3, DU145) PCa cell lines (Fig. 1D) using RTqPCR and western blot analysis. In the presence of androgen (charcoal‐stripped serum, CSS + 10 nm DHT), KLK14 mRNA was undetected in DU145 cells, detected at a low level in LNCaP cells, and at higher levels in C4‐2B and PC‐3 cells (+5.4‐ and +6.5‐fold compared to LNCaP cells, Fig. 1D Left). After androgen deprivation (AD; CSS + Veh, no DHT), KLK14 expression increased in LNCaP cells (+4.0 fold) but was not significantly modulated in C4‐2B, PC‐3, or DU145 cells (Fig. 1D Left). Western blot analysis confirmed KLK14 expression in the secretome of LNCaP cells after AD or ATT [using enzalutamide (Enz)] and high KLK14 expression in the more metastatic cell lines (C4‐2B and PC‐3 cells, Fig. 1D right). As expected, KLK3 was inversely regulated in LNCaP and C4‐2B cells, and not detected in PC‐3 cells (Fig. 1D right).

### 3.2 KLK14 overexpression in LNCaP cells leads to phenotypic changes

**Figure 2:**
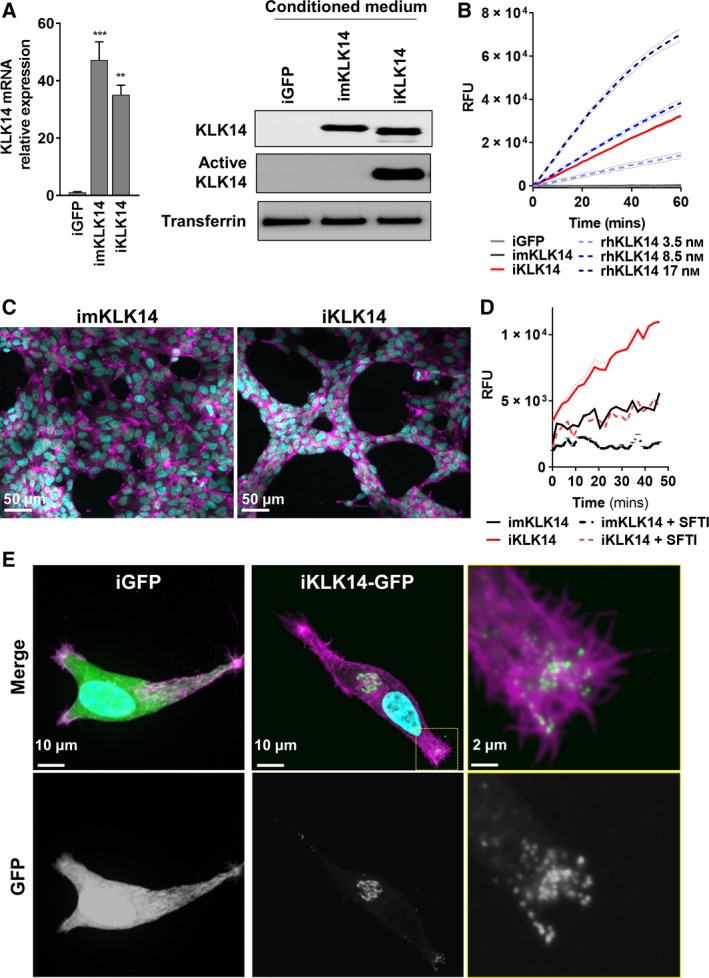
KLK14 proteolytic activity alters the morphology of LNCaP cells. (A) Left: KLK14 mRNA level determined by RTqPCR in LNCaP iGFP, imKLK14, and iKLK14 cells after 72 h of doxycycline treatment (mean ± SD). **P < 0.01, ***P < 0.001 compared to iGFP cells, two‐way ANOVA test. Right: Expression of KLK14 protein determined by western blot on concentrated CM. (B) KLK14 proteolytic activity in concentrated CM from iGFP‐, imKLK14‐, and iKLK14‐LNCaP cells compared to activity of active rhKLK14 (3.5, 8.5, and 17 nm) using a specific KLK14‐FRET peptide (mean ± SD). (C) Fluorescence microscopy imaging of imKLK14‐ and iKLK14‐LNCaP cells grown 3 days in serum‐free condition containing doxycycline and stained for F‐actin (phalloidin, purple) and nucleus (DAPI, blue). Scale bar: 50 µm. (D) KLK14 proteolytic activity in concentrated CM from imKLK14 and iKLK14‐LNCaP cells grown in serum‐free condition containing doxycycline ± selective KLK14 inhibitor (SFTI‐WCIR 2.5 µm) for 3 days (mean ± SD). (E) Fluorescence microscopy imaging of KLK14‐GFP and GFP in iKLK14‐GFP and iGFP ‐LNCaP cells costained for F‐actin (phalloidin, purple) and nucleus (DAPI, blue). Scale bar: 10 µm. GFP: green fluorescent protein; rhKLK14: Recombinant human KLK14; SFTI: sunflower trypsin inhibitor.

Our data demonstrate that KLK14 expression is elevated in PCa particularly in patients who develop CRPC, a lethal form of PCa. To decipher the molecular and biological roles of increased KLK14 expression in PCa cells, we expressed, under the control of a doxycycline‐inducible promoter, three variants in LNCaP cells: the wild‐type KLK14 (iKLK14), the catalytic‐mutant KLK14 (imKLK14), and the GFP sequence (iGFP). After 72h of doxycycline treatment, we observed a significant induction of KLK14 mRNA and protein in imKLK14‐ and iKLK14‐CM from LNCaP cells compared to iGFP‐LNCaP cells (Fig. 2A). Of note, in the iKLK14‐LNCaP cell CM, KLK14 was detected at a lower molecular weight compared to mKLK14 suggesting that KLK14 had been activated after secretion only in iKLK14‐LNCaP cells. Indeed, treatment of CM with a biotin‐tagged KLK14‐specific activity‐based probe (ABP, Fig. S1B, (Kasperkiewicz *et al.*, 2014)) and subsequent analysis by western blot using streptavidin revealed that only the iKLK14‐LNCaP cell line produced active KLK14 (Fig. 2A right). We also measured KLK14‐proteolytic activity in concentrated CM (50x) from iKLK14‐, imKLK14, and iGFP‐LNCaP cells using a KLK14‐specific FRET substrate (Fig. 2B). KLK14 activity was only detected in the CM from iKLK14‐LNCaP cells and was comparable to the activity of 8.5 nm active recombinant human KLK14 (rhKLK14) corresponding to a concentration of active KLK14 in iKLK14‐LNCaP cell CM of 170 pM. Interestingly, we observed that KLK14 expression modified the growth pattern of LNCaP cells with the formation of a trabecular network (Fig. 2C). Inhibition of KLK14‐proteolytic activity in iKLK14‐LNCaP cells using a selective reversible KLK14 inhibitor [sunflower trypsin inhibitor (SFTI)‐WCIR, (de Veer *et al.*, 2017), 2.5 µm, Fig. 2D] prevented the morphological change in LNCaP cells (Fig. S2A). Finally, live imaging of GFP‐tagged KLK14 in LNCaP cells (Fig. S2B, Video S1) showed that KLK14 transits via endoplasmic reticulum before being specifically localized to the lamellipodia and filopodia of PCa cells, whereas expression of GFP alone was distributed throughout the whole cell (Fig. 2E and Fig. S2C).

### 3.3 Identification of KLK14‐regulated proteins in the secretome of PCa cells

To identify the target substrates of KLK14 in PCa cells, we compared the secretome composition of iKLK14‐ and imKLK14‐LNCaP cells using the terminal amine isobaric‐tag labeling of substrates (TAILS) proteomic technique (Kleifeld *et al.*, 2011; Munasinghage Silva *et al.*, 2019). Through the depletion of trypsin‐generated neo‐N termini, TAILS analysis enables enrichment of N termini corresponding to natural protein N termini or to N termini generated by proteolytic processing.

**Figure 3:**
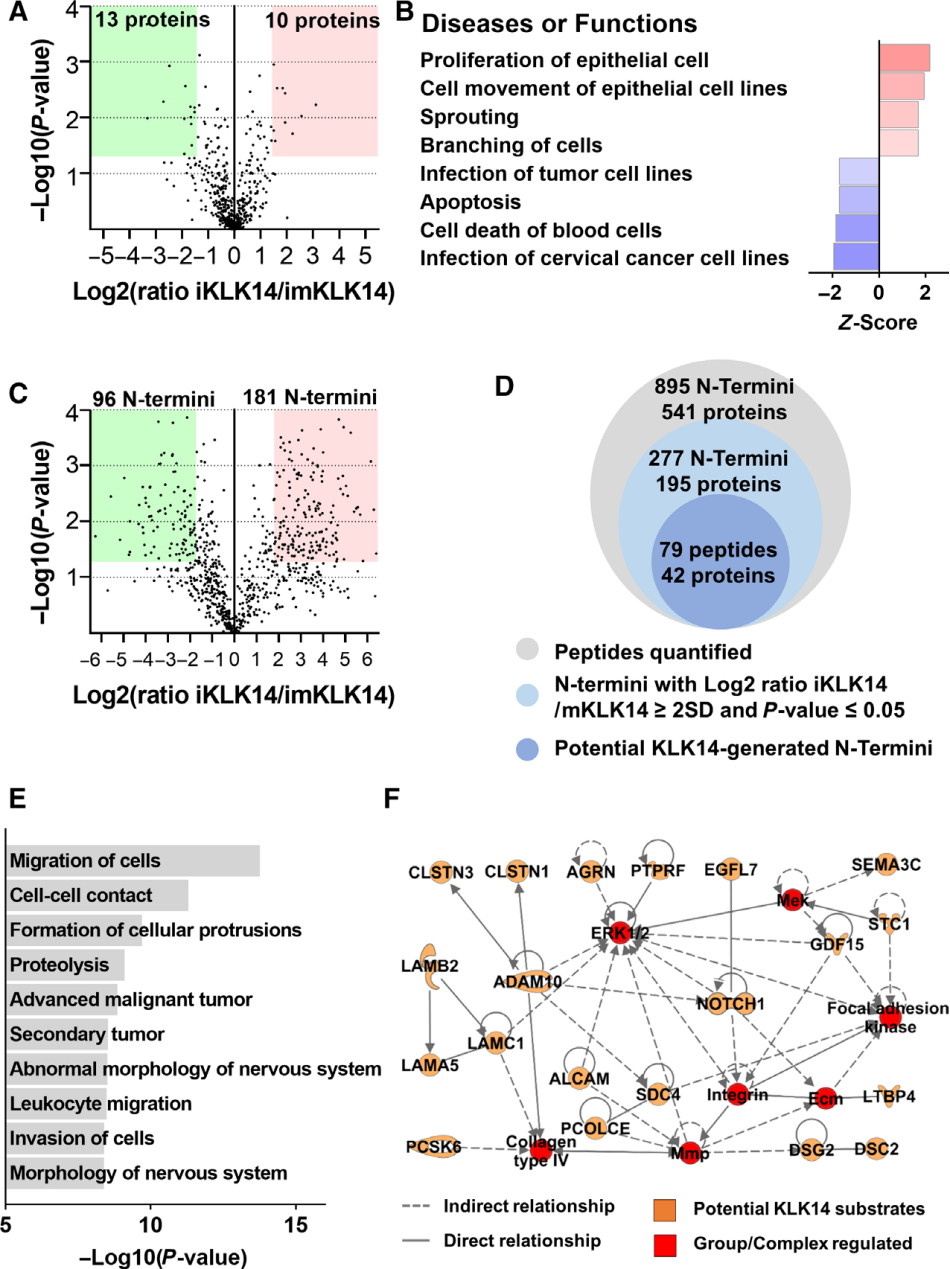
Identification of KLK14‐regulated proteins in secretome of LNCaP cells. (A) Volcano plot of *P*‐value [−Log10(*P*‐value)] as a function of FC [log2(ratio iKLK14/imKLK14)] for proteins identified in Pre‐TAILS analysis. The number of proteins with significant quantitative difference is indicated. (B) Diseases or functions predicted to be significantly activated or inhibited in IPA performed on proteins modulated after KLK14 induction in LNCaP cells. (C) Volcano plot of *P*‐value [−Log10(*P*‐value)] in function of FC [log2(ratio iKLK14/imKLK14)] for N termini identified in Pre‐TAILS and TAILS analysis. The number of peptides with significant quantitative difference is indicated. (D) Number of N termini quantified, significantly dysregulated, and corresponding to putative KLK14 substrates in Pre‐TAILS and TAILS analysis. (E) Diseases or functions found significantly enriched in IPA analysis performed on putative KLK14 substrates. (F) Interaction network identified by IPA composed of 20 out of 43 KLK14 substrates (indicated in orange in network).

The Pre‐TAILS analysis performed (which is similar to a shotgun whole secretome analysis (Munasinghage Silva *et al.*, 2019)) resulted in the identification and quantification of 2067 peptides [identified and quantified from at least two peptide‐to‐spectrum matches (PSMs), in at least two biological replicates and with a FDR < 0.01, Table S2] corresponding to 675 unique proteins (Table S3). Quantitative analysis demonstrated that only 23 of the 675 proteins identified (3.4% of quantified proteins, Fig. 3A and Table 1) had significant quantitative variations [log2 (ratio iKLK14/imKLK14) ≤ or ≥ 2SD, *P*‐value < 0.05 (Munasinghage Silva *et al.*, 2019), Fig. S3A,B] indicating that KLK14 does not drastically alter the expression profile of the secretome. As expected, considering the involvement of KLK14 in extracellular matrix (ECM) remodeling, components of the ECM represent the main class of proteins dysregulated including vitronectin (+3.1), AGRN (+2.6), laminin‐α5, β2, and γ1 (+1.6, +1.9, and +1.9, respectively) and fibronectin (+1.5). Also, several membrane proteins involved in cellular adhesion were modulated such as immunoglobulin superfamily member 8 (IGSF8) (+2.2), spondin‐2 (−1.5), and cadherin‐1 (−1.7; Table 1). Pathway analysis performed using ingenuity pathway analysis (IPA) (Kramer *et al.*, 2014) on KLK14‐modulated proteins indicated an activation of cellular functions related to cell motility and proliferation and an inhibition of cellular functions related to cell death or infection (Fig. 3B).

**Table 1:** Proteins dysregulated in the secretome of iKLK14 LNCaP cells identified in Pre‐TAILS. The list of proteins identified with significant quantitative differences between the secretome of iKLK14‐ and imKLK14‐LNCaP cells are summarized. Indicated are: UniProt accession; Gene symbol; Protein description; Protein coverage; Number of PSM and peptides; Log2(ratio iKLK14/imKLK14).

Accession	Symbol	Protein description	Coverage (%)	# PSMs	# Peptides	Log2 (iKLK14/imKLK14)
http://www.uniprot.org/uniprot/P04004	VTN	Vitronectin	10	18	5	3.1
http://www.uniprot.org/uniprot/O00468	AGRN	Agrin	35	147	53	2.6
http://www.uniprot.org/uniprot/Q969P0	IGSF8	Immunoglobulin superfamily member 8	15	23	8	2.2
http://www.uniprot.org/uniprot/P11047	LAMC1	Laminin subunit gamma‐1	37	141	45	1.9
http://www.uniprot.org/uniprot/P55268	LAMB2	Laminin subunit beta‐2	50	212	72	1.9
http://www.uniprot.org/uniprot/Q24JP5	TMEM132A	Transmembrane protein 132A	46	133	38	1.8
http://www.uniprot.org/uniprot/Q9Y5Y6	ST14	Suppressor of tumorigenicity 14 protein	42	77	24	1.6
http://www.uniprot.org/uniprot/O15230	LAMA5	Laminin subunit alpha‐5	43	287	104	1.6
http://www.uniprot.org/uniprot/P02751	FN	Fibronectin	31	132	49	1.5
http://www.uniprot.org/uniprot/Q9BXJ4	C1QTNF3	Complement C1q tumor necrosis factor‐related protein 3	8	3	2	1.5
http://www.uniprot.org/uniprot/P07910	HNRNPC	Heterogeneous nuclear ribonucleoproteins C1/C2	14	9	4	−1.5
http://www.uniprot.org/uniprot/Q9BUD6	SPON2	Spondin‐2	69	140	29	−1.5
http://www.uniprot.org/uniprot/Q7Z6Z7	HUWE1	E3 ubiquitin‐protein ligase HUWE1	1	6	3	−1.6
http://www.uniprot.org/uniprot/O75787	ATP6AP2	Renin receptor	50	34	11	−1.6
http://www.uniprot.org/uniprot/P25398	RPS12	40S ribosomal protein S12	40	13	4	−1.7
http://www.uniprot.org/uniprot/P12830	CDH1	Cadherin‐1	21	51	16	−1.7
http://www.uniprot.org/uniprot/Q02818	NUCB1	Nucleobindin‐1	43	40	18	−1.9
http://www.uniprot.org/uniprot/P09429	HMGB1	High mobility group protein B1	9	7	3	−1.9
http://www.uniprot.org/uniprot/P46060	RANGAP1	Ran GTPase‐activating protein 1	5	4	2	−1.9
http://www.uniprot.org/uniprot/Q9BRK5	SDF4	45 kDa calcium‐binding protein	44	42	14	−2.5
http://www.uniprot.org/uniprot/P16403	HIST1H1C	Histone H1.2	25	11	5	−2.7
http://www.uniprot.org/uniprot/Q9UII2	ATPIF1	ATPase inhibitor, mitochondrial	39	8	4	−3.3
http://www.uniprot.org/uniprot/P62633	CNBP	Cellular nucleic acid‐binding protein	27	12	4	−3.3

After enrichment of N Termini (TAILS analysis), 953 peptides were identified and quantified with 784 peptides corresponding to true N termini (Table S4). In comparison to our Pre‐TAILS analysis, the depletion of trypsin‐generated neo‐N termini using the HPG‐ALD polymer allowed a significant enrichment of protein N termini (82% vs 5%; Fig. 3C). Quantitative analysis performed on 895 N termini identified in Pre‐TAILS (111 N termini) and TAILS (784 N termini) experiments highlighted 277 N termini from 195 different proteins with significant quantitative variation [log2(ratio iKLK14/imKLK14) ≤ or ≥ 2SD, *P*‐value < 0.05, Fig. 3C and Table S5]. Due to the fact that KLK14 is a secreted protease with trypsin‐like activity, we decided to only consider as putative KLK14 substrates the 79 N‐terminal peptides with a P1 Lys or Arg and assigned to secreted or membrane‐bound proteins which represented 64 cleavage sites on 42 proteins (Fig. 3D, Table 2 and Table S6). Other N termini significantly modulated upon KLK14 expression, which could be due to an indirect effect of KLK14 through the regulation of the proteolytic activity of other proteases or protease inhibitors. The identified N Termini corresponding to intracellular proteins could correspond to proteins secreted via nonclassical secretion pathways or released into the CM by dead cells (Munasinghage Silva *et al.*, 2019).

**Table 2:** Putative KLK14 substrates and cleavage sites identified by TAILS. The list of putative KLK14 substrates identified in TAILS analysis are summarized. Indicated are: UniProt accession; gene symbol; protein description; position of cleavage site (P1 residue, *); Log2(ratio iKLK14/imKLK14), and the cellular location of the protein according to IPA. Proteins were classified in terms of their function according to available literature.

	Accession	Symbol	Protein description	P1	Log2 (FC)	Cellular location
Peptidases and peptidase regulators	http://www.uniprot.org/uniprot/P01034	CST3	Cystatin‐C	R 34	5.6	Extracellular space
	http://www.uniprot.org/uniprot/O14672	ADAM10	Disintegrin and metalloproteinase domain‐containing protein 10	R 448	2.2	Plasma membrane
	http://www.uniprot.org/uniprot/P00734	F2	Prothrombin	R 560	5.0	Extracellular space
	http://www.uniprot.org/uniprot/P07288	KLK3	Prostate‐specific antigen	R 24	2.7	Extracellular space
	http://www.uniprot.org/uniprot/Q9P0G3	KLK14	Kallikrein‐14	K 40	2.4	Extracellular space
				R 197	4.9	
				R 141	3.8	
	http://www.uniprot.org/uniprot/Q15113	PCOLCE	Procollagen C‐endopeptidase enhancer 1	R 149	2.3	Extracellular space
	http://www.uniprot.org/uniprot/P29122	PCSK6	Proprotein convertase subtilisin/kexin type 6	R 825	2.5	Extracellular space
	http://www.uniprot.org/uniprot/Q9Y5Y6	ST14	Suppressor of tumorigenicity 14 protein	R 614	4.0	Plasma membrane
				R 208	3.7	
	http://www.uniprot.org/uniprot/P10646	TFPI	Tissue factor pathway inhibitor	R 77	4.8	Extracellular space
ECM, proteoglycans, basement membrane constituents	http://www.uniprot.org/uniprot/O00468	AGRN	Agrin	R 261	3.3	Plasma membrane
				R 451	2.4	
				R 541	3.7	
				R 782	4.9	
				R 1810	2.5	
				R 1839	2.0	
				K 1863	3.5	
				R 1964	3.5	
	http://www.uniprot.org/uniprot/O15230	LAMA5	Laminin subunit alpha‐5	R 849	4.4	Extracellular space
				R 3582	3.9	
	http://www.uniprot.org/uniprot/P55268	LAMB2	Laminin subunit beta‐2	R 44	4.1	Extracellular space
				R 907	2.2	
	http://www.uniprot.org/uniprot/P11047	LAMC1	Laminin subunit gamma‐1	R 663	3.8	Extracellular space
	http://www.uniprot.org/uniprot/P31431	SDC4	Syndecan‐4	R 22	4.4	Plasma membrane
	http://www.uniprot.org/uniprot/Q9H3U7	SMOC2	SPARC‐related modular calcium‐binding protein 2	R 94	3.2	Extracellular space
Regulators of cell adhesion and morphology	http://www.uniprot.org/uniprot/O94910	ADGRL1	Adhesion G protein‐coupled receptor L1	R 37	4.4	Plasma membrane
	http://www.uniprot.org/uniprot/Q13740	ALCAM	CD166 antigen OS = Homo sapiens	R 344	3.7	Plasma membrane
	http://www.uniprot.org/uniprot/O94985	CLSTN1	Calsyntenin‐1	R 372	3.8	Plasma membrane
				R 665	6.3	
				K 791	3.1	
	http://www.uniprot.org/uniprot/Q9BQT9	CLSTN3	Calsyntenin‐3	R 151	3.5	Plasma membrane
	http://www.uniprot.org/uniprot/P98153	DGCR2	Integral membrane protein DGCR2/IDD	R 90	2.4	Plasma membrane
	http://www.uniprot.org/uniprot/Q02487	DSC2	Desmocollin‐2	R 190	2.6	
	http://www.uniprot.org/uniprot/Q14126	DSG2	Desmoglein‐2	K 215	5.2	Plasma membrane
				K 468	3.5	
	http://www.uniprot.org/uniprot/O15394	NCAM2	Neural cell adhesion molecule 2	R 118	6.6	Plasma membrane
				R 336	4.0	
	http://www.uniprot.org/uniprot/Q92859	NEO1	Neogenin	R 35	2.9	Plasma membrane
	http://www.uniprot.org/uniprot/O15031	PLXNB2	Plexin‐B2	R 807	2.9	Plasma membrane
	http://www.uniprot.org/uniprot/P10586	PTPRF	Receptor‐type tyrosine‐protein phosphatase F	R 282	4.4	Plasma membrane
				R 355	4.5	
				R 585	3.2	
				R 702	2.6	
	http://www.uniprot.org/uniprot/Q99985	SEMA3C	Semaphorin‐3C	R 55	2.2	Extracellular space
	http://www.uniprot.org/uniprot/Q9BUD6	SPON2	Spondin‐2	R 249	2.5	Extracellular space
	http://www.uniprot.org/uniprot/P50570	DNM2	Dynamin‐2	R 510	3.1	Plasma membrane
	http://www.uniprot.org/uniprot/Q969P0	IGSF8	Immunoglobulin superfamily member 8	R 94	3.7	Plasma membrane
Growth factors/ growth factor & hormone receptors and coreceptors	http://www.uniprot.org/uniprot/Q9NYQ6	CELSR1	Cadherin EGF LAG seven‐pass G‐type receptor 1	R 756	2.6	Plasma membrane
	http://www.uniprot.org/uniprot/Q9UHF1	EGFL7	Epidermal growth factor‐like protein 7	R 184	5.5	Extracellular space
	http://www.uniprot.org/uniprot/Q99988	GDF15	Growth/differentiation factor 15	R 217	4.6	Extracellular space
				R 107	3.9	
				R 263	3.1	
	http://www.uniprot.org/uniprot/Q8N2S1	LTBP4	Latent‐transforming growth factor beta‐binding protein 4	R 336	3.6	Extracellular space
	http://www.uniprot.org/uniprot/P46531	NOTCH1	Neurogenic locus notch homolog protein 1	R 207	4.4	Plasma membrane
Others	http://www.uniprot.org/uniprot/P02765	AHSG	Alpha‐2‐HS‐glycoprotein	K 131	6.1	Extracellular space
	http://www.uniprot.org/uniprot/P08758	ANXA5	Annexin A5	R 6	4.0	Plasma membrane
	http://www.uniprot.org/uniprot/Q13308	PTK7	Inactive tyrosine‐protein kinase 7	R 92	2.6	Plasma membrane
				R 226	2.7	
	http://www.uniprot.org/uniprot/Q9ULF5	SLC39A10	Zinc transporter ZIP10	R 41	1.9	Extracellular space
	http://www.uniprot.org/uniprot/P08294	SOD3	Extracellular superoxide dismutase [Cu‐Zn]	R 176	2.7	Extracellular space
	http://www.uniprot.org/uniprot/P5282	STC1	Stanniocalcin‐1	R 33	2.2	Extracellular space
	http://www.uniprot.org/uniprot/P02786	TFRC	Transferrin receptor protein 1	R 109	2.1	Plasma membrane

Among the putative KLK14 substrates (Table 2), several components of the proteolytic network were identified such as peptidases (ADAM metallopeptidase domain 10, ADAM10; prothrombin, F2; KLK3; KLK14, proprotein convertase subtilisin/kexin type 6, PCSK6; suppression of tumorigenicity 14 or matriptase, ST14) and peptidase modulators (peptidase inhibitors or enhancers, CST3; procollagen C‐endopeptidase enhancer; tissue factor pathway inhibitor). In accordance with the quantitative variations identified in our Pre‐TAILS analysis, several proteins involved in the formation of ECM and basement membranes were identified as KLK14 substrates (AGRN; laminin‐α5, LAMA5; laminin‐β2, LAMB2; laminin‐γ1, LAMC1; syndecan‐4, SDC4; SPARC‐related modular calcium binding 2, SMOC2). Moreover, the data indicated that KLK14 induces shedding of several membrane proteins that regulate cellular adhesion and cell‐to‐cell interactions, resulting in the release of soluble fragments in the extracellular space (adhesion G protein‐coupled receptor L1; CD166 antigen, ALCAM; DSG2; desmocollin‐2; neural adhesion molecule 2, IGSF8). The results also indicated roles for KLK14 in proteolytic processing of proteins involved in signal transduction (growth differentiation factor 15, epidermal growth factor‐like protein 7; latent‐transforming growth factor beta‐binding protein 4; neurogenic locus notch homolog protein 1).

Pathway analysis performed on putative KLK14 substrates indicated an enrichment in cellular functions related to cell migration, cell‐to‐cell contact, formation of cellular protrusions, and proteolysis (Fig. 3E). Additionally, the most significantly enriched mechanistic network involved 20 out of the 42 identified KLK14 substrates and important mediators of cellular migration (mitogen‐activated protein kinase 1, MAPK; integrins; and focal adhesion kinase, Fig. 3F).

**Figure 4:**
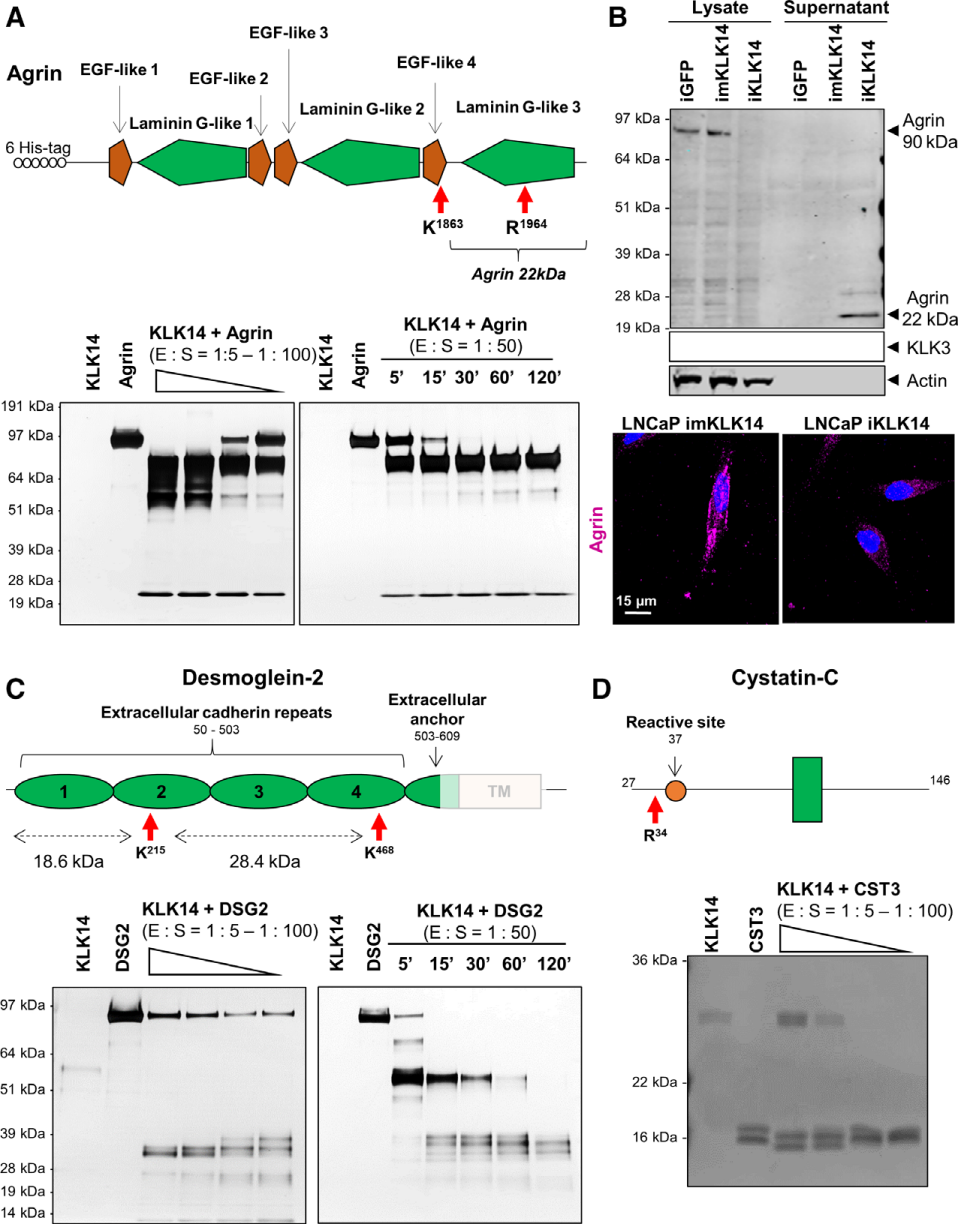
Validation of KLK14 newly identified substrates. (A) Top: Schematic of recombinant AGRN used with different protein domains and KLK14‐cleavage sites indicated. Bottom: Silver‐stained SDS/PAGE gels corresponding to the dose–response (left) and kinetics of recombinant AGRN proteolysis (right) by recombinant active KLK14. (B) Top: Western blot indicating the decrease of 90kDa AGRN in cell lysate and the release of 22kDa AGRN in the CM of iKLK14‐LNCaP cells. Bottom: Immunofluorescence microscopy imaging of imKLK14‐ and iKLK14‐LNCaP cells stained for AGRN (pink) and nucleus (DAPI, blue). Scale bar: 15 µm. (C) Top: Schematic of recombinant DSG2 used with its different protein domains and KLK14‐cleavage sites indicated. Bottom: Silver‐stained SDS/PAGE gels corresponding to the dose–response (left) and kinetics (right) of recombinant DSG2 proteolysis by recombinant active KLK14. (D) Top: Schematic of recombinant CST3 used with its different protein domains and KLK14‐cleavage sites indicated. Bottom: Silver‐stained SDS/PAGE gel corresponding to the dose–response of recombinant CST3 proteolysis by recombinant active KLK14. Dose–response proteolysis experiments have been performed at molar ratio of enzyme/substrates ranging from 1 : 5 to 1 : 100 for 2 h at 37 °C. Kinetic proteolysis experiments have been performed at a molar ratio of enzyme/substrate of 1 : 50 for 5, 15, 30, 60, and 120 min at 37 °C. EGF: epidermal growth factor; E:S: enzyme:substrate ratio.

To validate our TAILS analysis, we selected AGRN, DSG‐2, Cys‐C, and laminins for further validation in biochemical assays using recombinant or purified proteins. Our results showed that all proteins tested were direct KLK14 substrates (Fig. 4 and Fig. S3D) and were cleaved by KLK14 even at low enzyme : substrate ratios (E : S of 1 : 100) and after a short period of coincubation (5 min at E : S of 1 : 50, Fig. 4A,C,D).

Several identified KLK14 cleavage sites are known to generate bioactive molecules. For example, the cleavage after Lys40 of KLK14 (DENK^40^↓IIGG) results in removal of the KLK14 propeptide (Borgono *et al.*, 2007b), thus enabling the conversion of pro‐KLK14 to active KLK14, and confirming the ability of KLK14 to autoactivate. Similarly, the cleavage site corresponding to the conversion of pro‐KLK3/PSA to active KLK3/PSA (ILSR^24^↓IVGG) was also enriched upon KLK14 expression, confirming that KLK14 is a natural activator of pro‐KLK3 (Yoon *et al.*, 2007). Numerous KLK14 cleavage sites have been identified on the proteoglycan AGRN (Arg^261^, Arg^451^, Arg^541^, Arg^782^, Arg^1810^, Arg^1839^, Lys^1863^, and Arg^1964^), suggesting KLK14‐mediated degradation of this protein. Particularly, the cleavage site after Lys^1863^ results in the generation of the bioactive 22 kDa AGRN fragment, which positively regulates filopodia formation (Matsumoto‐Miyai *et al.*, 2009). Of note, in biochemical assays, KLK14 generated the 22 kDa AGRN fragment very efficiently and western blot analysis performed on whole cell lysates and CM from LNCaP cells showed that overexpression of KLK14 led to a decrease of 90 kDa AGRN in LNCaP cell lysate and the release of the 22 kDa AGRN fragment in the supernatant (Fig. 4B top). Furthermore, immunofluorescence staining also showed a decrease of cell‐bound AGRN in LNCaP iKLK14 cells compared to LNCaP imKLK14 cells (Fig. 4B bottom).

### 3.4 Impact of KLK14 on the transcriptome of LNCaP cells

**Figure 5:**
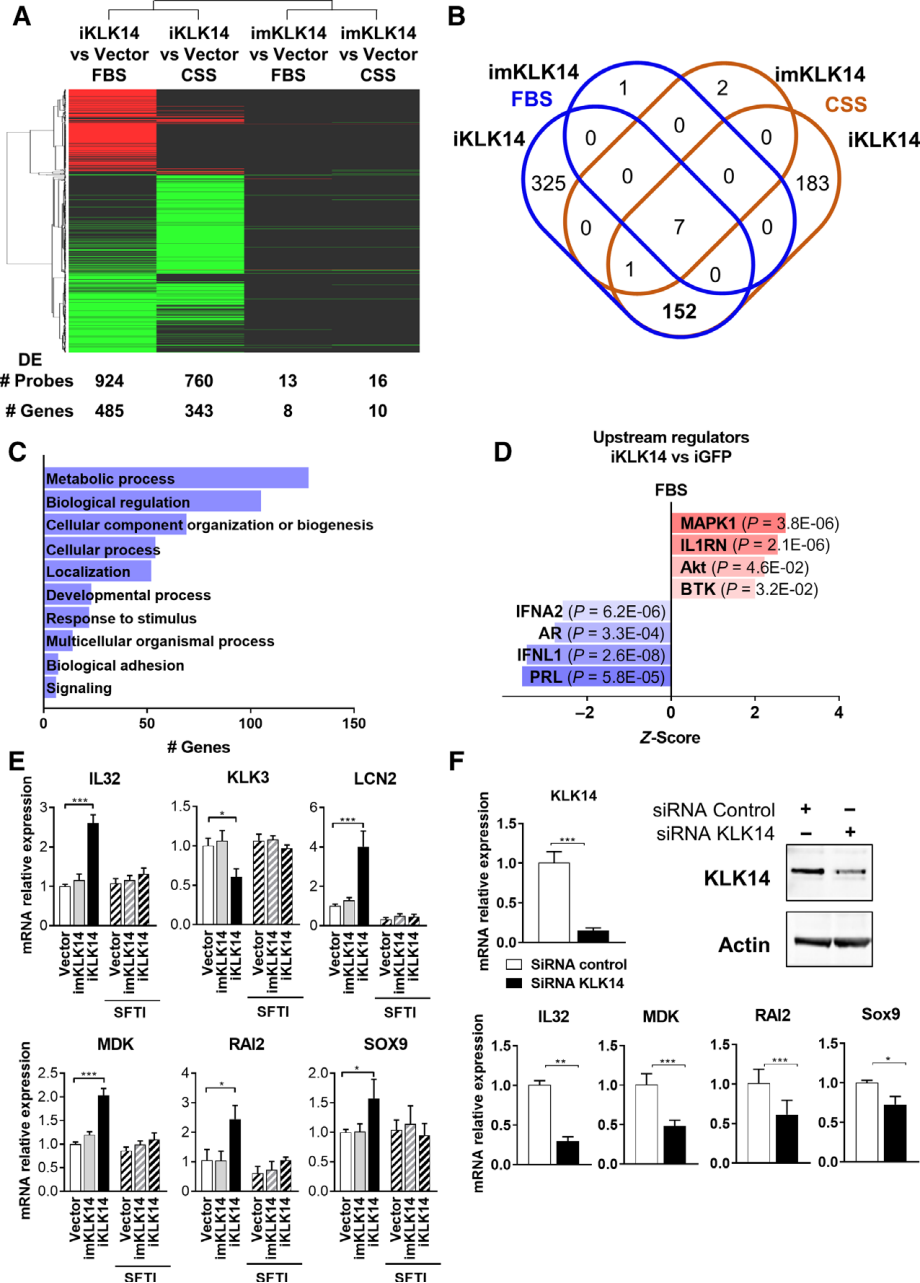
Identification of KLK14‐induced transcriptome modulations in PCa cells. (A) Heat‐map representing all the probes significantly dysregulated (FC ≥ or ≤ to 1.5, *P*‐value ≤ 0.05) in the transcriptomic analysis (green = downregulated; red = upregulated). The number of probes and genes dysregulated in each comparison is indicated. (B) Venn diagram representing the overlap between the genes dysregulated in imKLK14‐ and iKLK14‐LNCaP cells compared to vector‐only LNCaP cells in FBS and CSS. (C) Cellular function enrichment analysis performed using Panther/Gene ontology on genes dysregulated after KLK14 induction in the FBS condition. (D) Upstream regulators analysis from IPA analysis performed on genes dysregulated after KLK14 induction in FBS condition. (E) Expression of IL32, KLK3, LCN2, MDK, RAI2, and SOX9 (mRNA level, RTqPCR, mean ± SD) in iGFP‐, imKLK14‐ and iKLK14‐LNCaP cells grown in RPMI‐1% FBS containing doxycycline ± SFTI‐WCIR (2.5 µm) for 3 days. (F) Expression KLK14, IL32, MDK, RAI2 and Sox9 (mRNA level, RTqPCR, mean ± SD) in PC3 cells transfected with control or KLK14‐siRNA grown in RPMI‐1% FBS for 3 days. Protein KLK14 expression determined by western blot is presented. *N* = 3, *P < 0.05, **P < 0.01, ***P < 0.001 in Two‐way ANOVA test.

To determine the consequences of KLK14 expression on the transcriptome of PCa cells, we performed gene expression analysis in iKLK14‐, imKLK14‐, and iGFP‐LNCaP cells using a custom Agilent 180 k probe RNA microarray (Wang *et al.*, 2018). This experiment was performed in both 1% FBS and 1% CSS in order to determine if KLK14 exerts different effects in the presence or absence of androgens. Using a FC cut off in probe intensity of 1.5 and *P*‐value cut off of 0.05 compared to the control condition (iGFP in FBS or CSS), a minimal effect on the transcriptome of LNCaP cells was observed following mKLK14 induction with only 13 and 16 probes significantly dysregulated in FBS and CSS conditions, respectively, corresponding to 8 and 10 genes (Fig. 5A and Table S7). However, overexpression of KLK14 had a major impact on the LNCaP cell transcriptome with 924 and 760 probes significantly dysregulated in FBS and CSS conditions, respectively, corresponding to 485 and 343 genes (Fig. 5A and Table S7). We observed that 160 genes were commonly dysregulated in both FBS and CSS conditions after KLK14 overexpression (Fig. 5B). The list of the top 15 genes commonly up‐ or downregulated upon KLK14 expression in FBS and CSS condition is presented in Table 3. To validate the microarray gene expression analysis, dysregulation of 12 genes was confirmed by RTqPCR (Fig. S4A). Gene enrichment analysis performed using the Panther Classification System highlighted a significant enrichment in genes involved in ‘metabolic processes’, ‘biological regulation’, ‘cellular component organization and biogenesis’, and ‘cellular process’ (Fig. 5C). We used IPA to determine which upstream signaling pathways could be involved in the KLK14‐mediated transcriptome modulation (Fig. 5D). Interestingly, the most significant mediator predicted to be activated upon KLK14 expression was MAPK1 which was also identified as being regulated by KLK14 in our proteomic analysis. Other signaling molecules predicted to be activated after KLK14 expression were: interleukin 1 (IL1) receptor antagonist, protein kinase B (Akt), and Bruton's tyrosine kinase. Conversely, interferon alpha‐2, AR, interferon lambda 1, and prolactin are predicted to be inactivated (Fig. 5D).

**Table 3:** Top 15 genes up‐ and downregulated upon KLK14 expression. The top 15 genes commonly identified as up‐ or downregulated in iKLK14‐LNCaP cells compared to vector‐only LNCaP cells in both FBS and CSS conditions are summarized. Indicated are: gene symbol (ID); FC; *P*‐value; Entrez gene name.

ID	FBS		CSS		Entrez Gene Name
	FC	*P*‐value	FC	*P*‐value	
IL32	2.35	<0.001	2.86	<0.001	Interleukin 32
LCN2	2.2	0.011	2.1	<0.001	Lipocalin 2
AOC1	1.98	<0.001	1.67	<0.001	Amine oxidase, copper containing 1
PDZK1IP1	1.86	<0.001	1.86	<0.001	PDZK1 interacting protein 1
CCDC80	1.84	<0.001	1.52	<0.001	Coiled‐coil domain containing 80
RAI2	1.78	<0.001	1.6	<0.001	Retinoic acid induced 2
PIGR	1.77	<0.001	2.08	<0.001	Polymeric immunoglobulin receptor
MDK	1.73	<0.001	1.8	<0.001	Midkine (neurite growth‐promoting factor 2)
KCNMB4	1.7	<0.001	1.66	<0.001	Potassium calcium‐activated channel subfamily M regulatory beta subunit 4
PLA2G2A	1.7	<0.001	1.61	<0.001	Phospholipase A2 group IIA
PLD1	1.65	<0.001	1.5	0.001	Phospholipase D1
IL1B	1.63	<0.001	1.5	<0.001	Interleukin 1 beta
CFB	1.59	<0.001	1.89	<0.001	Complement factor B
HOXA11‐AS	1.59	<0.001	1.58	<0.001	HOXA11 antisense RNA
TMEM74B	1.57	<0.001	2.03	<0.001	Transmembrane protein 74B
IFIT1	−1.86	<0.001	−1.84	<0.001	Interferon induced protein with tetratricopeptide repeats 1
KLK2	−1.9	<0.001	−1.94	<0.001	Kallikrein‐related peptidase 2
MCM3AP	−1.93	0.001	−1.86	0.002	Minichromosome maintenance complex component 3 associated protein
HYOU1	−1.94	<0.001	−1.57	0.006	Hypoxia upregulated 1
KLK3	−1.94	<0.001	−2.3	<0.001	Kallikrein‐related peptidase 3
MYH9	−1.96	0.002	−1.97	0.002	Myosin, heavy chain 9, nonmuscle
SMC1A	−1.96	0.001	−2.05	0.001	Structural maintenance of chromosomes 1A
CDCP1	−2.03	<0.001	−2	<0.001	CUB domain‐containing protein 1
GOLGA3	−2.03	<0.001	−2.02	<0.001	Golgin A3
GCN1L1	−2.08	0.001	−2.2	<0.001	GCN1, eIF2 alpha kinase activator homolog
NCOR2	−2.11	<0.001	−1.97	0.001	Nuclear receptor corepressor 2
ATP2A2	−2.2	<0.001	−2.11	0.001	ATPase sarcoplasmic/endoplasmic reticulum Ca^2+^ transporting 2
PLOD2	−3.18	<0.001	−2	0.001	Procollagen‐lysine,2‐oxoglutarate 5‐dioxygenase 2
NDUFA4L2	−3.72	<0.001	−2.33	0.001	NADH dehydrogenase (ubiquinone) 1 alpha subcomplex, 4‐like 2
UTRN	−4.15	0.049	−1.68	<0.001	Utrophin

Finally, we demonstrated that the dysregulation of IL32, KLK3, lipocalin‐2 (LCN2), midkine (MDK), retinoid acid induced‐2 (RAI2), and SRY‐Box 9 (Sox9) was dependent on KLK14 proteolytic activity as any changes were totally abrogated in the presence of a KLK14‐selective reversible inhibitor (SFTI‐WCIR, 2.5 µm, Fig. 5E). In addition, treatment of PC‐3 cells with KLK14 siRNA led to an inverse regulation of IL32, MDK, midasin (MDN1), nuclear receptor corepressor‐2 (NCOR2), RAI2, and Sox9 expression (Fig. 5F and Fig. S4B).

### 3.5 Biological function of KLK14 in PCa cells

Our proteomic and transcriptomic results indicated the involvement of KLK14 in the regulation of the MAPK pathway and cellular adhesion and migration. To validate our findings, we performed several biological assays to assess the level of MAPK activation in PCa cells upon KLK14 expression as well as the impact of KLK14 on cellular migration.

**Figure 6:**
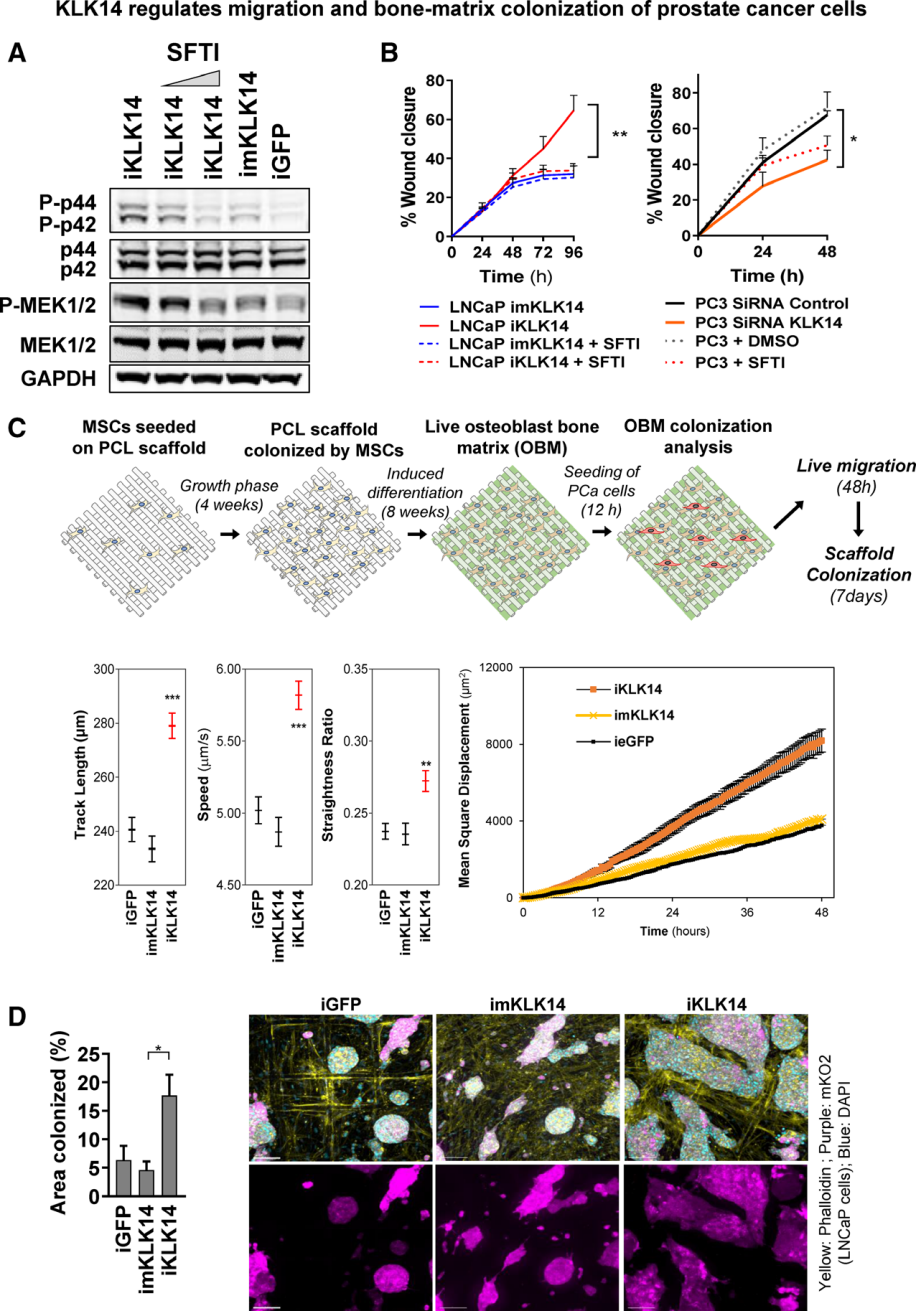
KLK14 regulates MAPK activation, migration and bone‐matrix colonization of PCa cells. (A) Analysis of phosphorylation states of MAPK pathway proteins (p42/44 and Mek1/2) in iGFP‐, imKLK14‐, and iKLK14‐LNCaP cells grown in RPMI‐1% FBS for 3 days in the presence of doxycycline ± 1 or 2.5 µm SFTI‐WCIR. *N* = 3; representative blot shown. (B) Scratch wound migration assay performed with imKLK14‐ and iKLK14‐LNCaP cells in same conditions as that in A (left) and with PC‐3 cells transfected with siRNA nontargeting and siRNA KLK14 in RPMI‐1% FBS ± 2.5 µm SFTI‐WCIR (right). Results are presented as mean ± SD. *N* = 3, *P < 0.05, **P < 0.01, Two‐way ANOVA test. (C) Top: Schematic picturing the workflow of production of human OBM and migration/colonization assays. Bottom: Results from live cell migration analysis of iGFP‐, imKLK14‐ and iKLK14‐LNCaP cells onto OBM in RPMI + 1%FBS containing doxycycline with measured average track length (µm), speed (µm·s^−1^), straightness ratio, and MSD (µm^2^). Measurements performed on an average of 315 cells per condition (*N* = 2 independent biological replicates with two different OBM preparations). Results are presented as mean ± SEM. (D) Measurement of OBM scaffold colonization by iGFP‐, imKLK14‐, and iKLK14‐LNCaP cells 7 days after seeding. Left: Graphic representing the OBM scaffold area occupied by LNCaP cells (% of whole scaffold surface, mean ± SD). Right: Representative images of iGFP‐, imKLK14‐ and iKLK14‐LNCaP cells on OBM scaffold after 7 days of culture in RPMI‐1% FBS containing doxycycline (*N* = 4 scaffolds per condition). Scale bar: 500 µm.

First, KLK14‐mediated activation of MAPK in PCa cells was confirmed in iKLK14 LNCaP cells (Fig. 6A). Indeed, we showed that the level of MAPK activation (phosphorylation of p44/42 and MEK1/2) was significantly elevated in iKLK14‐LNCaP cells compared to iGFP‐ or imKLK14‐LNCaP cells. Moreover, MAPK activation was dependent on the proteolytic activity of KLK14 as treatment with high concentration of the KLK14‐selective inhibitor diminished the effect (SFTI‐WCIR, 2.5 µm, Fig. 6A and Fig. S5A).

Secondly, we assessed the effect of KLK14 expression on migration of PCa cells. Using a live cell migration assay, we identified that iKLK14‐LNCaP cells had a higher rate of migration compared to imKLK14‐LNCaP cells (Fig. 6B left). Additionally, we demonstrated that this promigratory effect was directly mediated by KLK14‐proteolytic activity since it was reversed by the KLK14‐selective inhibitor SFTI‐WCIR (2.5 µm, Fig. 6B left). Accordingly, knockdown of KLK14 expression in PC‐3 cells or treatment with KLK14‐specific inhibitor also reduced cell migration (Fig. 6B right).

Finally, because KLK14 was found to be elevated in PCa metastases compared to primary prostate tumors (Fig. 1B) and PCa primarily metastasizes to bone forming mostly osteoblastic lesions, we also performed a migration assay with iGFP‐, imKLK14‐, and iKLK14‐LNCaP cells on a novel OBM live model mimicking osteoblastic mineralized tissue from the bone microenvironment ((Bock *et al.*, 2019), Fig. 6C top panel). In this setting, we confirmed that KLK14 expression significantly increased the migration of LNCaP cells compared to expression of GFP or mKLK14 with a significant increase in both speed and straightness (i.e., persistence) of cancer cell displacement, leading to an overall increase in track length and MSD (Fig. 6C bottom panel). In addition, we also assessed the long‐term growth of LNCaP cells on the OBM microtissues. After 10 days, LNCaP cells expressing KLK14 colonized the microtissues to a greater extent compared to cells expressing GFP or mKLK14 (Fig. 6D left panel). Specifically, LNCaP cells expressing iGFP or imKLK14 colonized 7 and 4% of the entire OBM surface, respectively (purple staining corresponding to mKO2 expression), whereas cells overexpressing KLK14 colonized 17% of the OBM microtissues. Imaging analysis of the fixed scaffolds at the end point revealed that iKLK14 LNCaP cells spread more and formed larger colonies on the OBM microtissues, compared to iGFP and imKLK14 LNCaP cells which grew as smaller clusters (Fig. 6D right panel and Fig. S5B). Overall, results demonstrated that KLK14‐expression positively impacted on the colonization of PCa in a bone‐like microenvironment, suggesting that KLK14 could be involved in bone microenvironment remodeling, which is a necessary step for the formation of effective PCa metastases in bone.

## 4 Discussion

This study highlights that KLK14 expression is elevated in high Gleason score PCa and in PCa‐derived metastases, as well as a negative association between KLK14 expression levels and DFP confirming previous reports suggesting an association between KLK14 and PCa aggressiveness (Lose *et al.*, 2012; Rabien *et al.*, 2008; Rose *et al.*, 2018; Yousef *et al.*, 2003). Additionally, we identified for the first time that KLK14 protein expression is reduced in patients treated and responsive to NHT and that high KLK14 expression is more frequently observed in patients developing CRPC compared to NHT responders. This indicates that KLK14 expression is reactivated in PCa cells which escape ATT and is increased in metastatic PCa, suggesting that the expression levels of KLK14 in PCa tumors or serum could be informative to predict treatment efficacy or aggressiveness of prostate tumors.

The involvement of KLK14 in cancer progression has been reported in multiple studies; however, our work is the first to combine multiple omics and functional approaches to decipher the molecular and biological function of this protease. Our functional assays clearly showed a direct role for KLK14 in the regulation of cohesion and migration of PCa cells. The first hint about the function of KLK14 in PCa cells comes from its localization at the cell lamellipodia which plays an important role in the control of cellular motility (Innocenti, 2018). Additionally, we observed that increased KLK14 proteolytic activity led to a modification of cell pattern with the formation of a tubular network composed of cohesive cells. Of note, it has been previously proposed that PCa cells invade surrounding tissues through the formation of a cohesive cell network rather than as a single cell (Nagle and Cress, 2011). Accordingly, results from migration assays performed with PCa cells overexpressing or silenced for KLK14 as well as using a KLK14‐selective inhibitor confirmed the involvement of KLK14 in the control of PCa cell migration.

The determination of a protease substratome is the key to identify the molecular events under its control and in fine its biological function. To date, KLK14 substrates are limited to several proteins identified by substrate prediction or by biochemical assays but which have not been confirmed in a complex cellular environment (Borgono *et al.*, 2007a; Borgono *et al.*, 2007b; Lawrence *et al.*, 2010; Rajapakse and Takahashi, 2007; de Veer *et al.*, 2012). Our cell‐based proteomics analysis confirmed the proteolytic activity of KLK14 toward several known substrates (laminin subunit proteins: LAMA5, LAMB2, and LAMC1; KLK3/PSA; and KLK14) and identified 37 new putative KLK14 substrates. The majority of KLK14 substrates identified are involved in the regulation of cell adhesion, migration, and morphology and have previously been found to be involved in progression of PCa. For example, DSG‐2 is a protein involved in desmosome formation which has been recently proposed as a marker for aggressive PCa (Barber *et al.*, 2014). Two other KLK14 substrates, IGSF8 and SEMA3C, are involved in the regulation of cellular morphology and regulate androgen‐independent growth and metastasis of PCa cells (Levina *et al.*, 2015; Tam *et al.*, 2017; Zhang *et al.*, 2003). Several other newly identified substrates are involved in the formation of the ECM, such as AGRN, SDC4, and SMOC2 (Shvab *et al.*, 2016; Wang *et al.*, 2015). Particularly, the heparan sulfate proteoglycan (HSPG) AGRN is a critical regulator for the progression of several cancers, particularly through its involvement in cancer cell migration and shedding (Chakraborty *et al.*, 2015; Nagarajan *et al.*, 2018). Interestingly, we showed that KLK14 generates a bioactive AGRN 22 kDa fragment which plays an essential role in the filopodia‐inducing effect of AGRN (Matsumoto‐Miyai *et al.*, 2009). Finally, our analysis also demonstrated that KLK14 regulates the activity of other proteases such as ADAM10 and ST14/matriptase. Specifically, we identified two KLK14 cleavage sites in ST14/matriptase (Arg208 and Arg614) localized between its transmembrane domain and its catalytic site indicating that KLK14 controls the shedding of matriptase which has been previously reported as an important mechanism in invasion of PCa cells (Cheng *et al.*, 2013; Wu *et al.*, 2017). Additionally, because matriptase was reported as one of the activators of pro‐KLKs present in the epidermidis (Sales *et al.*, 2010), its shedding by KLK14 could be of high importance in the regulation of the epidermal proteolytic cascade involved in skin desquamation.

Finally, our study is the first to date to determine the impact of KLK14 on the transcriptome of PCa cells. By comparison of the effect of wild‐type active and catalytically mutant KLK14, we were able to demonstrate that KLK14’s impact on the PCa cell transcriptome was mainly mediated through its proteolytic activity. Importantly, several of the genes found to be regulated by KLK14 have previously been associated with PCa progression. For example, LCN2 is known to be involved in the regulation of PCa cell proliferation, migration, and invasion as well as in progression toward CRPC (Ding *et al.*, 2015; Ding *et al.*, 2016; Tung *et al.*, 2013). MDK expression is associated with advanced PCa such as the neuroendocrine phenotype and CRPC (Nordin *et al.*, 2013; You *et al.*, 2008) as well as a stem‐cell phenotype in PCa (Erdogan *et al.*, 2017). The pathway analysis performed on the KLK14‐mediated transcriptome regulation predicted an activation of the MAPK pathway and an inactivation of the AR axis, both of which play essential roles in advanced PCa. The activation of MAPK (particularly the ERK/MEK pathway) is associated with the development of CRPC and decreased survival for PCa patients (Mukherjee *et al.*, 2011; Rodriguez‐Berriguete *et al.*, 2012). The activation of the AR axis is generally considered as a requirement for PCa progression even after development of CRPC when PCa cells do not require the presence of androgens to grow and survive (Einstein *et al.*, 2019). However, it is increasingly accepted that AR‐negative subpopulations of PCa are present in prostate tumors such as neuroendocrine populations or stem‐cell‐like PCa cells. Recently, it has been demonstrated that certain subpopulations of metastatic PCa cells do not express the AR and do not present neuroendocrine features. Interestingly, it has been shown that these ‘double‐negative’ PCa cells are particularly sensitive to MAPK inhibitors (Bluemn *et al.*, 2017). It is plausible that KLK14 could be a significant modulator in these scenarios. The activity of trametinib (a MEK1/2 inhibitor) in metastatic castration‐resistant PCa is currently assessed in clinical trial (NCT02881242).

Our study supports the functional involvement of KLK14 in progression of PCa and suggests that targeting KLK14 could reduce the aggressiveness of PCa cells and limit tumor progression. Several synthetic KLK inhibitors have already been produced by modifying natural protease inhibitors (Di Paolo *et al.*, 2019; Goettig *et al.*, 2010). The most potent and selective KLK14 inhibitor developed to date is a bioengineered version of the SFTI‐WCIR which has a Ki of 7 nm for KLK14 and provides over 1000‐fold selectivity against proteases related by function (KLK5 and KLK7) and structure (trypsin) (de Veer *et al.*, 2017). However, because SFTI‐based inhibitors are small, peptidic molecules with very short serum half‐life, further inhibitor optimization is needed to enable their use in preclinical models. In this study, we also used a recently developed activity‐based probe by combining an electrophilic diphenyl phosphonate warhead with an amino acid sequence compatible with the specificity of KLK14 (YASR). This covalent molecule (*K*_obs_/*I* = 451 ± 19 m ^−1·^S^−1^) is a prototype that enables rapid and accurate detection of KLK14 activity *in vitro*. Our current studies are focused on incorporating unique unnatural amino acids into this peptidic‐warhead scaffold in order to develop an *in vivo* compatible KLK14 ABP/inhibitor with optimal properties (potency, selectivity, and serum half‐life) that will be employed in preclinical testing.

## 5 Conclusion

To conclude, our study reinforced the evidence for the involvement of KLK14 in the development of advanced forms of PCa and highlights the molecular mechanisms involved in the protumorigenic effect of KLK14 in PCa. Further studies are required to establish the links between the KLK14 substratome and KLK14‐regulated genes/pathways as well as to determine which molecular events are the key in the protumorigenic effect of KLK14 *in vivo*. In the future, agents specifically targeting KLK14 could be beneficial for PCa patients by limiting the development of aggressive forms of this disease.

## Conflict of interest

The authors declare no conflict of interest.

## Author contributions

The experimental design was by TK, NB, CN, JDH, and JAC. TK, NB, SL, AR, JP, CES, LF, and LMS performed the experiments presented, analyzed the data, and prepared figures for the manuscript. ML, SS, EDW, MG, JB, CN, EWT, and JH significantly contributed in the interpretation of data. SL and EWT designed and produced KLK14‐ABP. JH produced and characterized KLK14 inhibitor. AL produced KLK14‐selective FRET substrate. All authors contributed to writing and editing this manuscript.

## Supporting information


**Fig. S1.** (A) Examples of KLK14 staining in prostate tumors. Scale bar = 50 µm. (B) Structure of KLK14 activity‐based probe used. DPP: diphenyl phosphonate.

**Fig. S2.** (A) Representative bright‐field images of LNCaP cells imKLK14 and iKLK14 stimulated with doxycycline in RPMI + 1% FBS for 72 h in presence of DMSO or KLK14‐Specific inhibitor (SFTI‐WCIR, 2.5 µm). Scale bar = 100 µm. (B) Western blot analysis for KLK14 and GFP expression in concentrated CM from LNCaP cells imKLK14, imKLK14‐GFP, iKLK14 and iKLK14‐GFP. (C) Fluorescence microscopy imaging of KLK14‐GFP and GFP (Green) in iKLK14‐GFP and iGFP‐LNCaP cells costained for F‐actin (phalloidin, purple) and nucleus (DAPI, blue). Scale bar: 15 µm.

**Fig. S3.** (A) Distribution of fold‐change values (log2(ratio iKLK14/imKLK14)) with SD for the proteins (left) or peptides (right) identified in Pre‐TAILS analysis. (B) Volcano plot of p‐value (‒Log10(p‐value)) in function of fold‐change (log2(ratio iKLK14/imKLK14)) for peptides identified in Pre‐TAILS analysis. Number of peptides with significant quantitative difference are indicated. (C) Summary of peptides identified with an unmodified N terminus, a TMT‐labeled N terminus or an acetylated N terminus identified in Pre‐TAIL and TAILS analysis. (D) Western blot analysis for Laminin‐alpha 5 (Green) and gamma‐1 (red) in samples from the dose‐response proteolysis of basement membrane proteins by recombinant active KLK14.

**Fig. S4.** (A) Expression of IL32, KLK3, LCN2, CFB, MDK, RAI2, SOX9, PlGR, KCNMB4, KLK2, GPR158 and PDZK1IP1 (mRNA level, RTqPCR, mean ± SD) in iGFP‐, imKLK14‐ and iKLK14‐LNCaP cells grown in RPMI‐1% FBS or 1% CSS for 3 days. (B) Expression of KCNMB4, LCN2, MDN1, NCOR2 and TMEM74B (mRNA level, RTqPCR, mean ± SD) in PC3 cells transfected with control or KLK14‐siRNA grown in RPMI‐1% FBS for 3 days. *N *= 3, *P < 0.05, **P < 0.01, ***P < 0.001; Two‐way ANOVA test.

**Fig. S5.** (A) Densitometry analysis performed on western blots for the analysis of phosphorylation‐states of MAPK pathway proteins (p42/44 and Mek1/2) in iGFP‐, imKLK14‐ and iKLK14‐LNCaP cells grown in RPMI‐1% FBS for 3 days in presence of doxycycline ± 1 or 2.5 µm SFTI‐WCIR. *N *= 3, mean ± SD, *P < 0.05, **P < 0.01, Two‐way ANOVA test. (B) Fluorescence images showing the colonization of OBM micro‐tissues by iGFP‐, imKLK14‐ and iKLK14‐LNCaP cells.

**Table S1.** List of primers used in RTqPCR experiments.
**Table S2.** List of proteins identified and quantified in Pre‐TAILS analysis.
**Table S3.** List of peptides identified and quantified in Pre‐TAILS analysis.
**Table S4.** List of peptides identified and quantified in TAILS analysis.
**Table S5.** List of N Termini with significant variation between secretome of LNCaP cells imKLK14 and iKLK14.
**Table S6.** List of N Termini potentially generated by KLK14.
**Table S7.** List of genes significantly deregulated at least in one condition in the transcriptome analysis.

**Video S1.** Live cell imaging of iKLK14‐GFP (Green) in iKLK14‐GFP‐LNCaP cells costained for cytoplasm (cell tracker, Gray) and nucleus (Hoechst, Blue). Time lapse between each frame is 4 min.

**Video S2.** Live cell imaging of iKLK14‐GFP (Green) in iKLK14‐GFP‐LNCaP cells costained for cytoplasm (cell tracker, Gray) and nucleus (Hoechst, Blue). Time lapse between each frame is 4 min.
