# Supplementary figures and images for: The molecular function of kallikrein‐related peptidase 14 demonstrates a key modulatory role in advanced prostate cancer
Source: Mol Oncol. 2019 Nov 28;14(1):105–28. doi: 10.1002/1878-0261.12587 (PMC6944120; doi:10.1002/1878-0261.12587)

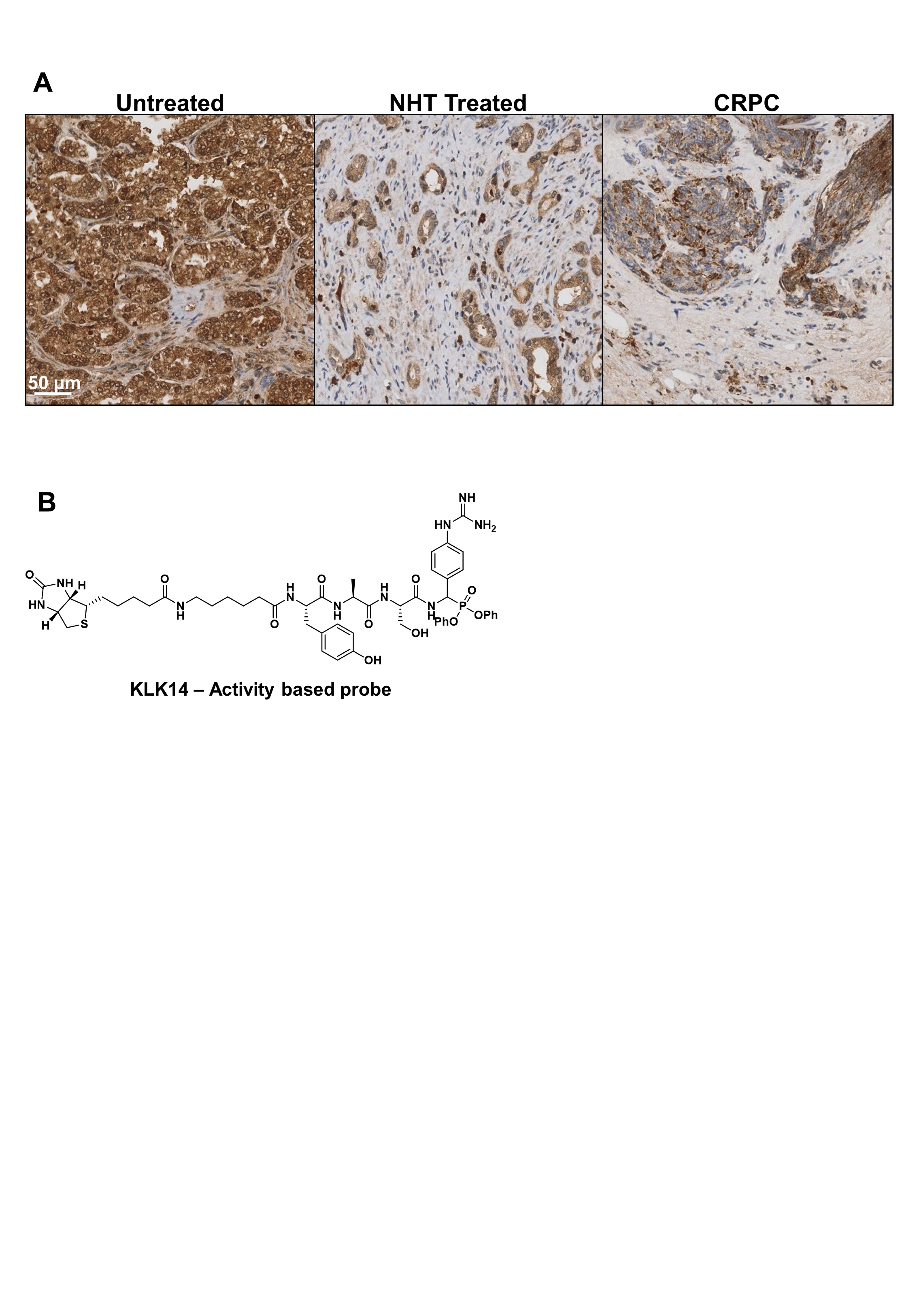

Supplement: Supplementary file 1 — Fig. S1. (A) Examples of KLK14 staining in prostate tumors. Scale bar = 50 µm. (B) Structure of KLK14 activity‐based probe used. DPP: diphenyl phosphonate. [file MOL2-14-105-s001.TIF]

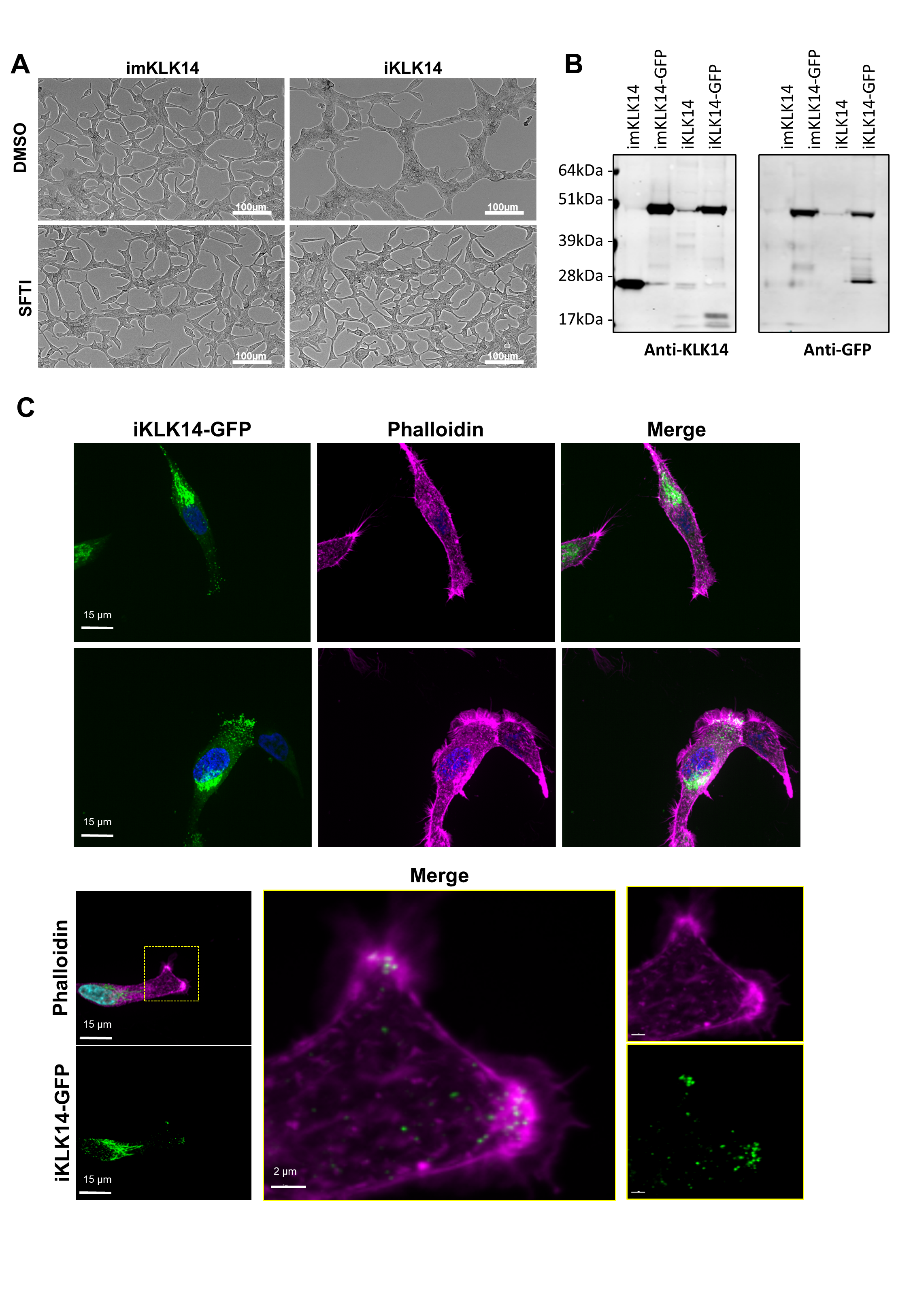

Supplement: Supplementary file 2 — Fig. S2. (A) Representative bright‐field images of LNCaP cells imKLK14 and iKLK14 stimulated with doxycycline in RPMI + 1% FBS for 72 h in presence of DMSO or KLK14‐Specific inhibitor (SFTI‐WCIR, 2.5 µm). Scale bar = 100 µm. (B) Western blot analysis for KLK14 and GFP expression in concentrated CM from LNCaP cells imKLK14, imKLK14‐GFP, iKLK14 and iKLK14‐GFP. (C) Fluorescence microscopy imaging of KLK14‐GFP and GFP (Green) in iKLK14‐GFP and iGFP‐LNCaP cells costained for F‐actin (phalloidin, purple) and nucleus (DAPI, blue). Scale bar: 15 µm. [file MOL2-14-105-s002.TIF]

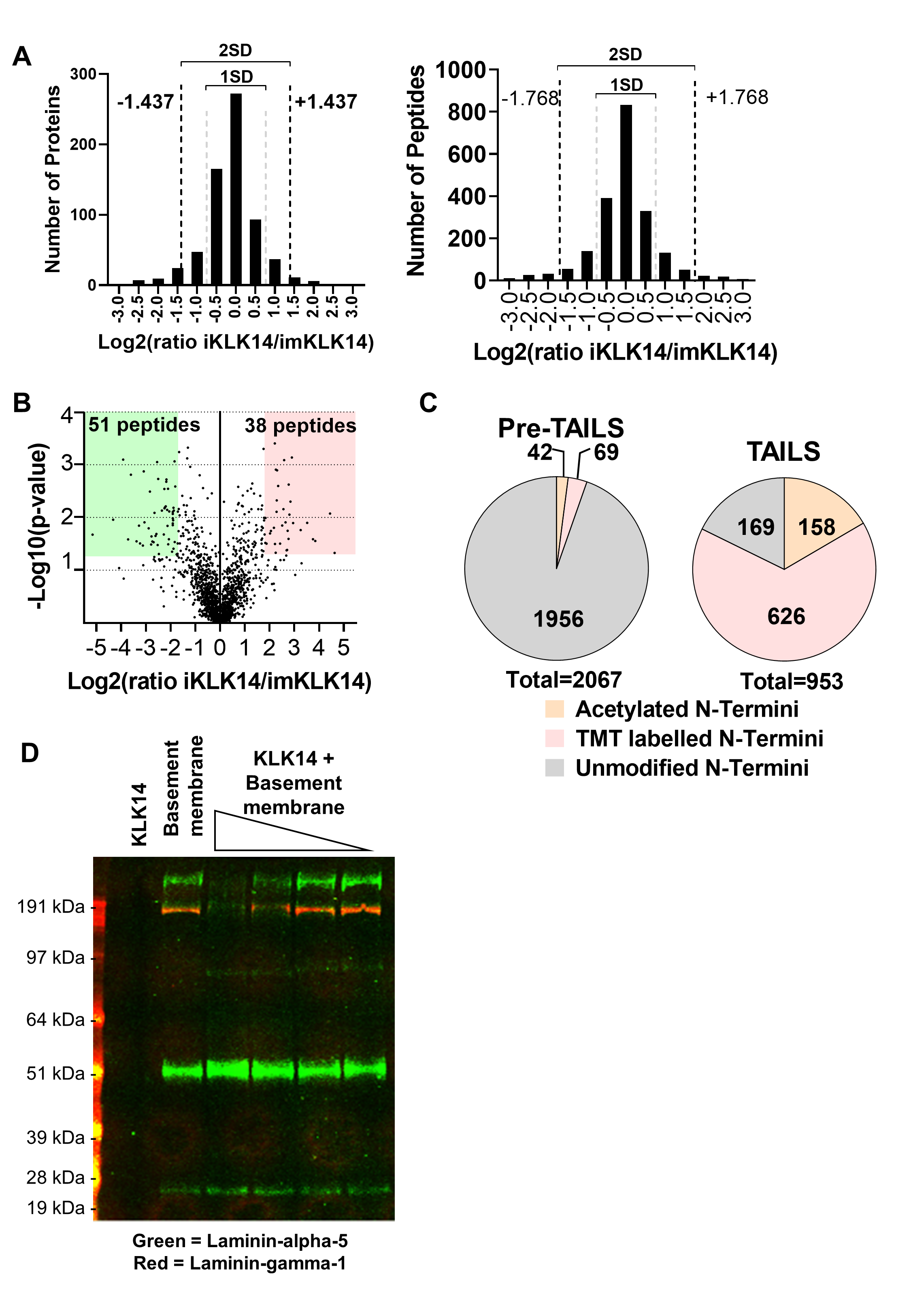

Supplement: Supplementary file 3 — Fig. S3. (A) Distribution of fold‐change values (log2(ratio iKLK14/imKLK14)) with SD for the proteins (left) or peptides (right) identified in Pre‐TAILS analysis. (B) Volcano plot of p‐value (‒Log10(p‐value)) in function of fold‐change (log2(ratio iKLK14/imKLK14)) for peptides identified in Pre‐TAILS analysis. Number of peptides with significant quantitative difference are indicated. (C) Summary of peptides identified with an unmodified N terminus, a TMT‐labeled N terminus or an acetylated N terminus identified in Pre‐TAIL and TAILS analysis. (D) Western blot analysis for Laminin‐alpha 5 (Green) and gamma‐1 (red) in samples from the dose‐response proteolysis of basement membrane proteins by recombinant active KLK14. [file MOL2-14-105-s003.tif]

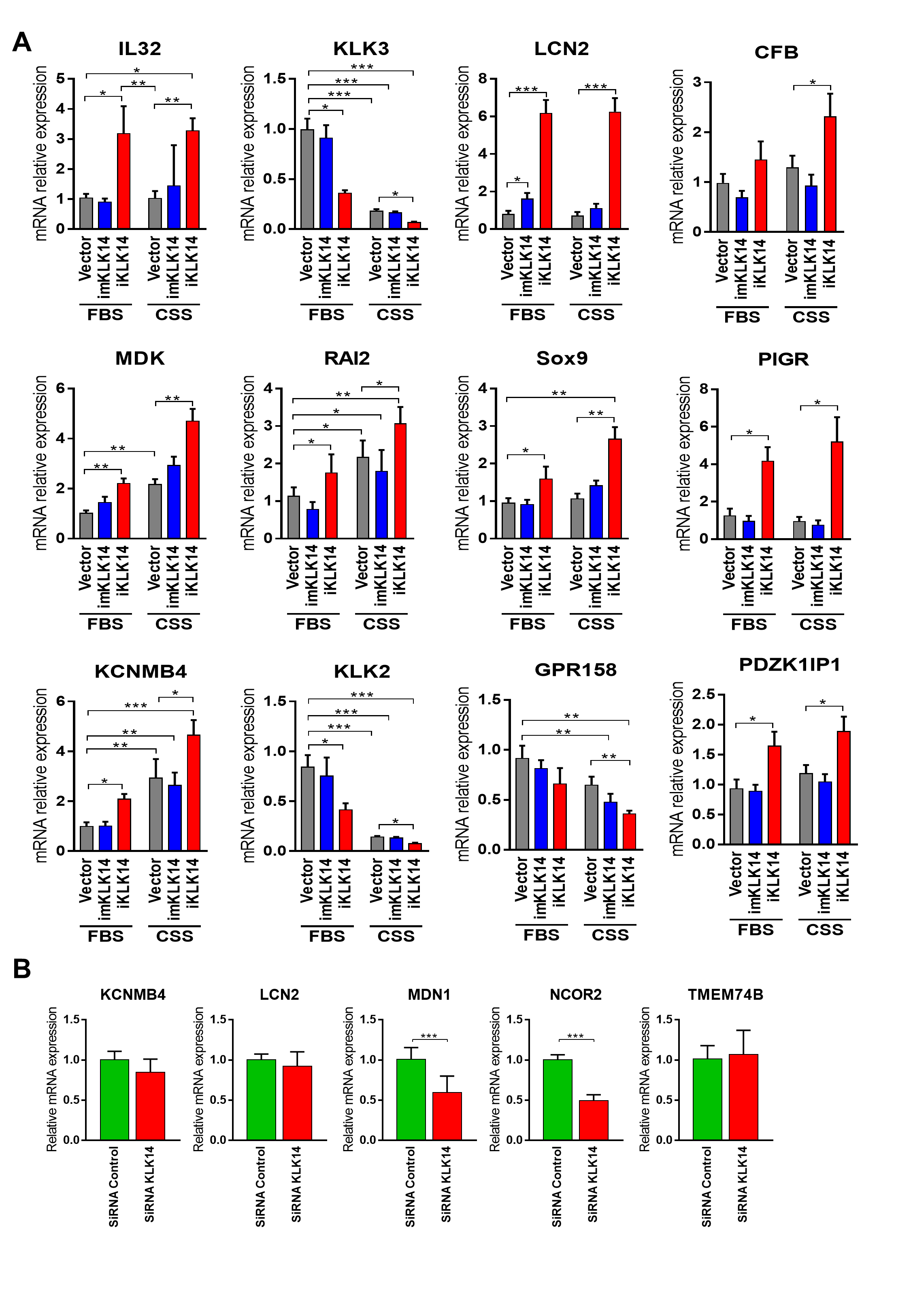

Supplement: Supplementary file 4 — Fig. S4. (A) Expression of IL32, KLK3, LCN2, CFB, MDK, RAI2, SOX9, PlGR, KCNMB4, KLK2, GPR158 and PDZK1IP1 (mRNA level, RTqPCR, mean ± SD) in iGFP‐, imKLK14‐ and iKLK14‐LNCaP cells grown in RPMI‐1% FBS or 1% CSS for 3 days. (B) Expression of KCNMB4, LCN2, MDN1, NCOR2 and TMEM74B (mRNA level, RTqPCR, mean ± SD) in PC3 cells transfected with control or KLK14‐siRNA grown in RPMI‐1% FBS for 3 days. N = 3, *P < 0.05, **P < 0.01, ***P < 0.001; Two‐way ANOVA test. [file MOL2-14-105-s004.TIF]

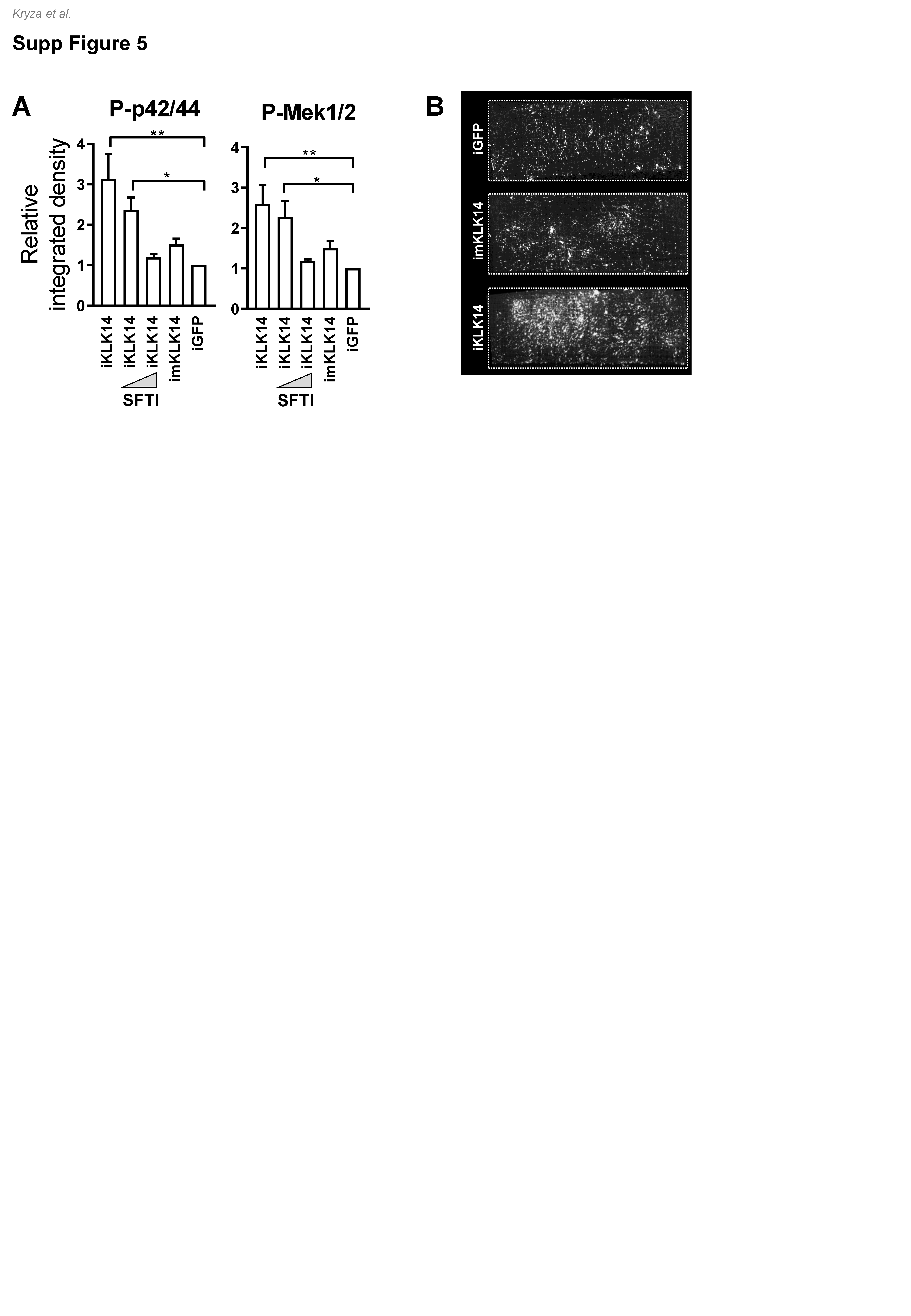

Supplement: Supplementary file 5 — Fig. S5. (A) Densitometry analysis performed on western blots for the analysis of phosphorylation‐states of MAPK pathway proteins (p42/44 and Mek1/2) in iGFP‐, imKLK14‐ and iKLK14‐LNCaP cells grown in RPMI‐1% FBS for 3 days in presence of doxycycline ± 1 or 2.5 µm SFTI‐WCIR. N = 3, mean ± SD, *P < 0.05, **P < 0.01, Two‐way ANOVA test. (B) Fluorescence images showing the colonization of OBM micro‐tissues by iGFP‐, imKLK14‐ and iKLK14‐LNCaP cells. [file MOL2-14-105-s005.TIF]
