# Supplementary material for: The molecular function of kallikrein‐related peptidase 14 demonstrates a key modulatory role in advanced prostate cancer
Source: Mol Oncol. 2019 Nov 28;14(1):105–28. doi: 10.1002/1878-0261.12587 (PMC6944120; doi:10.1002/1878-0261.12587)
Supplement: Supplementary file 7 — Video S1. Live cell imaging of iKLK14‐GFP (Green) in iKLK14‐GFP‐LNCaP cells costained for cytoplasm (cell tracker, Gray) and nucleus (Hoechst, Blue). Time lapse between each frame is 4 min. [file MOL2-14-105-s007.pptx]

## Slide 1
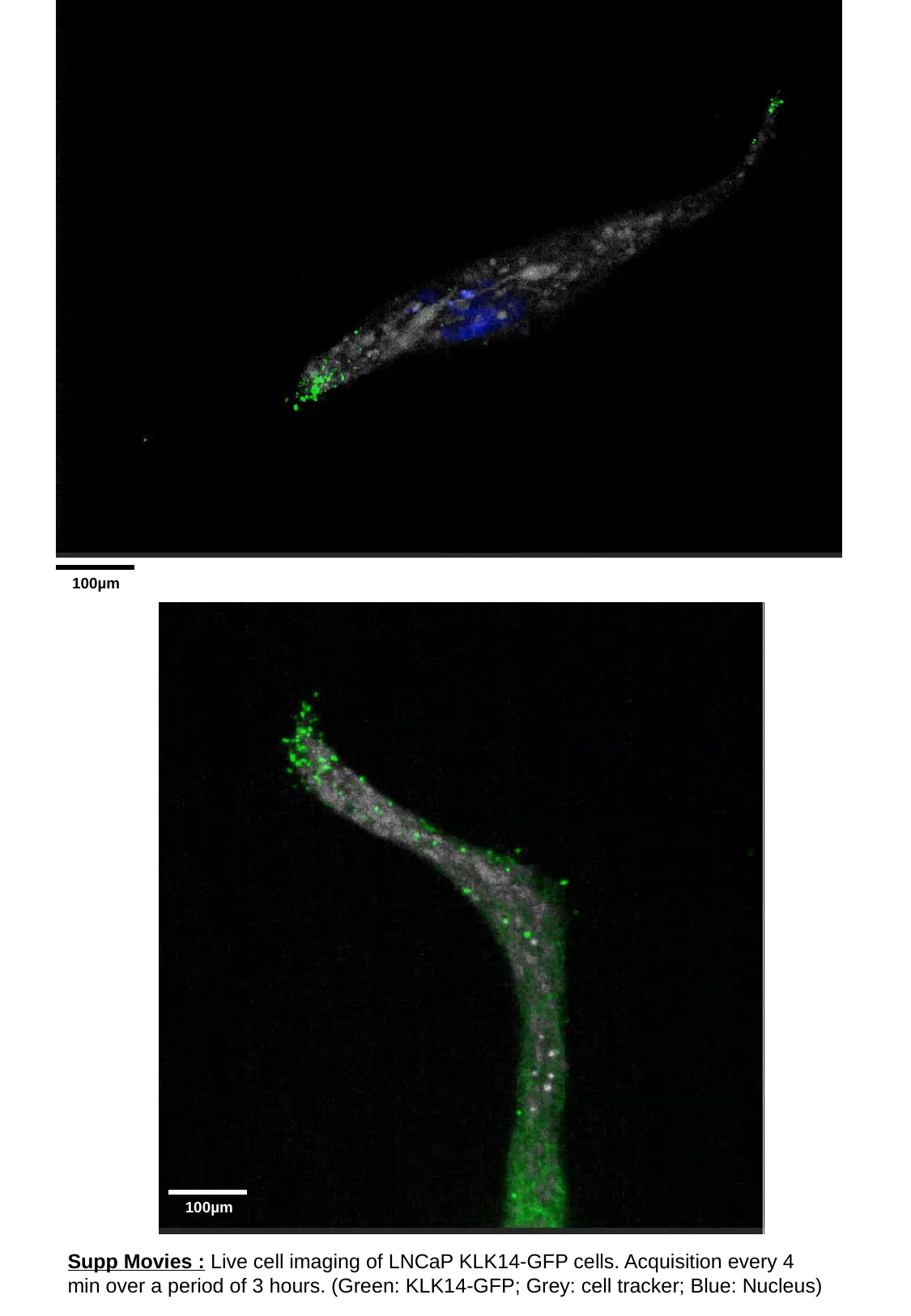

100µm
100µm
Supp Movies : Live cell imaging of LNCaP KLK14-GFP cells. Acquisition every 4 min over a period of 3 hours. (Green: KLK14-GFP; Grey: cell tracker; Blue: Nucleus)
