# Supplementary material for: The molecular function of kallikrein‐related peptidase 14 demonstrates a key modulatory role in advanced prostate cancer
Source: Mol Oncol. 2019 Nov 28;14(1):105–28. doi: 10.1002/1878-0261.12587 (PMC6944120; doi:10.1002/1878-0261.12587)
Supplement: Supplementary file 9 [file MOL2-14-105-s009.docx]

**Figure S1: A)** Examples of KLK14 staining in prostate tumors. Scale bar = 50 µm. **B)** Structure of KLK14 activity-based probe used. DPP: diphenyl phosphonate.

**Figure S2: A)** Representative bright-field images of LNCaP cells imKLK14 and iKLK14 stimulated with doxycycline in RPMI + 1% FBS for 72h in presence of DMSO or KLK14-Specific inhibitor (SFTI-WCIR, 2.5 µM). Scale bar =100 µm. **B)** Western blot analysis for KLK14 and GFP expression in concentrated CM from LNCaP cells imKLK14, imKLK14-GFP, iKLK14 and iKLK14-GFP. **C)** Fluorescence microscopy imaging of KLK14-GFP and GFP (Green) in iKLK14-GFP and iGFP-LNCaP cells costained for F-actin (phalloidin, purple) and nucleus (DAPI, blue). Scale bar: 15 µm. GFP: Green fluorescent protein; rhKLK14: Recombinant human KLK14; SFTI: Sunflower Trypsin inhibitor.

**Figure S3: A)** Distribution of fold-change values (log2(ratio iKLK14/imKLK14)) with SD for the proteins (left) or peptides (right) identified in Pre-TAILS analysis. **B)** Volcano plot of p-value (-Log10(p-value)) in function of fold-change (log2(ratio iKLK14/imKLK14)) for peptides identified in Pre-TAILS analysis. Number of peptides with significant quantitative difference are indicated. **C)** Summary of peptides identified with an unmodified N terminus, a TMT-labeled N terminus or an acetylated N terminus identified in Pre-TAIL and TAILS analysis. **D)** Western blot analysis for Laminin-alpha 5 (Green) and gamma-1 (red) in samples from the dose-response proteolysis of basement membrane proteins by recombinant active KLK14.

**Figure S4: A)** Expression of IL32, KLK3, LCN2, CFB, MDK, RAI2, SOX9, PlGR, KCNMB4, KLK2, GPR158 and PDZK1IP1 (mRNA level, RTqPCR, mean ± SD) in iGFP-, imKLK14- and iKLK14-LNCaP cells grown in RPMI-1% FBS or 1% CSS for 3 days. **B)** Expression of KCNMB4, LCN2, MDN1, NCOR2 and TMEM74B (mRNA level, RTqPCR, mean ± SD) in PC3 cells transfected with control or KLK14-siRNA grown in RPMI-1% FBS for 3 days. N=3, *p<0.05, **p<0.01, ***P<0.001; Two-way ANOVA test.

**Figure S5: A)** Densitometry analysis performed on western blots for the analysis of phosphorylation-states of MAPK pathway proteins (p42/44 and Mek1/2) in iGFP-, imKLK14- and iKLK14-LNCaP cells grown in RPMI-1% FBS for 3 days in presence of doxycycline ± 1 or 2.5 µM SFTI-WCIR. N=3, , mean ± SD, *p<0.05, **p<0.01, Two-way ANOVA test. **B)** Fluorescence images showing the colonization of OBM micro-tissues by iGFP-, imKLK14- and iKLK14-LNCaP cells.

**Video S1 and S2:** Live cell imaging of iKLK14-GFP (Green) in iKLK14-GFP-LNCaP cells costained for cytoplasm (cell tracker, Gray) and nucleus (Hoechst, Blue). Time lapse between each frame is 4 min.

**Table S1: List of primers used in RTqPCR experiments**

**Table S2: List of proteins identified and quantified in Pre-TAILS analysis.** Proteins with significant quantitative variation are shown in bold. Indicated are: UniProt accession; Protein description; Cellular location (according to IPA); Molecular Class (according to IPA); q-value combined; Sum PEP score; Protein coverage; Number of peptides and PSM; Number of unique peptides; Amino acid (AA) length of identified protein; Molecular weight of identified protein (MW); calculated isoelectric point (calc. pI); Identification score (Sequest HT algorithm); number of razor peptides; Log2(ratio iKLK14/imKLK14), -Log10(p-value), Confidence of protein identification in each sample.

**Table S3: List of peptides identified and quantified in Pre-TAILS analysis.** Peptides with significant quantitative variation are indicated in bold. Indicated are: Peptide sequence with preceding and following amino acid; Peptide modification; Modification detected in master protein; Quality PEP and q-value; Number of protein groups associated to identified peptide; Number of proteins associated to identified peptide; Number of PSM; UniProt accession for assigned master protein; Description of assigned master protein; Cellular location and Molecular Class (according to IPA) of assigned protein; Position of identified peptide on assigned master protein; Number of missed cleavages; Theoretical MH+ mass (Da); Log2(ratio iKLK14/imKLK14), -Log10(p-value), Confidence of protein identification in each sample; Percolator q-value; Percolator PEP; XCorr

**Table S4: List of peptides identified and quantified in TAILS analysis.** Peptides with significant quantitative variation are indicated in bold. Indicated are: Peptide sequence with preceding and following amino acid; Peptide modification; Modification detected in master protein; Quality PEP and q-value; Number of protein groups associated to identified peptide; Number of proteins associated to identified peptide; Number of PSM; UniProt accession for assigned master protein; Description of assigned master protein; Cellular location and Molecular Class (according to IPA) of assigned protein; Position of identified peptide on assigned master protein; Number of missed cleavages; Theoretical MH+ mass (Da); Log2(ratio iKLK14/imKLK14), -Log10(p-value), Confidence of protein identification in each sample; Percolator q-value; Percolator PEP; XCorr.

**Table S5: List of N Termini with significant variation between secretome of LNCaP cells imKLK14 and iKLK14.** Indicated are: Peptide sequence with preceding and following amino acid; Peptide modification; Modification detected in master protein; Quality PEP and q-value; Number of protein groups associated to identified peptide; Number of proteins associated to identified peptide; Number of PSM; UniProt accession for assigned master protein; Description of assigned master protein; Cellular location and Molecular Class (according to IPA) of assigned protein; Position of identified peptide on assigned master protein; Number of missed cleavages; Theoretical MH+ mass (Da); Log2(ratio iKLK14/imKLK14), -Log10(p-value), Confidence of protein identification in each sample; Percolator q-value; Percolator PEP; XCorr.

**Table S6: List of N Termini potentially generated by KLK14.** Indicated are: Peptide sequence with preceding and following amino acid; Peptide modification; Modification detected in master protein; Quality PEP and q-value; Number of protein groups associated to identified peptide; Number of proteins associated to identified peptide; Number of PSM; UniProt accession for assigned master protein; Description of assigned master protein; Cellular location and Molecular Class (according to IPA) of assigned protein; Position of identified peptide on assigned master protein; Number of missed cleavages; Theoretical MH+ mass (Da); Log2(ratio iKLK14/imKLK14), -Log10(p-value), Confidence of protein identification in each sample; Percolator q-value; Percolator PEP; XCorr.

**Table S7: List of genes significantly deregulated at least in one condition in the transcriptome analysis.** Indicated are: Gene ID, Fold change (FC), p-value, Entrez gene name, Cellular location and Molecular class according to IPA.
